# Toxicogenomic response of *Pseudomonas aeruginosa *to ortho-phenylphenol

**DOI:** 10.1186/1471-2164-9-473

**Published:** 2008-10-10

**Authors:** Chantal W Nde, Hyeung-Jin Jang, Freshteh Toghrol, William E Bentley

**Affiliations:** 1 Center for Biosystems Research, University of Maryland Biotechnology Institute, College Park, Maryland 20742, USA; 2 Microarray Research Laboratory, Biological and Economic Analysis Division, Office of Pesticide Programs, U. S. Environmental Protection Agency, Fort Meade, Maryland 20755, USA

## Abstract

**Background:**

*Pseudomonas aeruginosa *(*P. aeruginosa*) is the most common opportunistic pathogen implicated in nosocomial infections and in chronic lung infections in cystic fibrosis patients. Ortho-phenylphenol (OPP) is an antimicrobial agent used as an active ingredient in several EPA registered disinfectants. Despite its widespread use, there is a paucity of information on its target molecular pathways and the cellular responses that it elucidates in bacteria in general and in *P. aeruginosa *in particular. An understanding of the OPP-driven gene regulation and cellular response it elicits will facilitate more effective utilization of this antimicrobial and possibly lead to the development of more effective disinfectant treatments.

**Results:**

Herein, we performed a genome-wide transcriptome analysis of the cellular responses of *P. aeruginosa *exposed to 0.82 mM OPP for 20 and 60 minutes. Our data indicated that OPP upregulated the transcription of genes encoding ribosomal, virulence and membrane transport proteins after both treatment times. After 20 minutes of exposure to 0.82 mM OPP, genes involved in the exhibition of swarming motility and anaerobic respiration were upregulated. After 60 minutes of OPP treatment, the transcription of genes involved in amino acid and lipopolysaccharide biosynthesis were upregulated. Further, the transcription of the ribosome modulation factor (*rmf*) and an alternative sigma factor (*rpo*S) of RNA polymerase were downregulated after both treatment times.

**Conclusion:**

Results from this study indicate that after 20 minutes of exposure to OPP, genes that have been linked to the exhibition of anaerobic respiration and swarming motility were upregulated. This study also suggests that the downregulation of the *rmf *and *rpoS *genes may be indicative of the mechanism by which OPP causes decreases in cell viability in *P. aeruginosa*. Consequently, a protective response involving the upregulation of translation leading to the increased synthesis of membrane related proteins and virulence proteins is possibly induced after both treatment times. In addition, cell wall modification may occur due to the increased synthesis of lipopolysaccharide after 60 minutes exposure to OPP. This gene expression profile can now be utilized for a better understanding of the target cellular pathways of OPP in *P. aeruginosa *and how this organism develops resistance to OPP.

## Background

Hospital-acquired infections caused by opportunistic pathogens present a serious threat to public health. Nosocomial infections are estimated to occur in 5% of all acute-care hospitalizations and in more than 2 million cases each year [1]. *P. aeruginosa *is the most common opportunistic pathogen responsible for hospital acquired burn wound infections, urinary tract infections and ventilator-associated pneumonia [2-5]. In cystic fibrosis patients, *P. aeruginosa *is implicated in chronic lung infections, leading to high rates of illness and death [6]. In the increasing AIDS population, 50% of deaths have been linked *P. aeruginosa *bacteremia [7]. The increasing prevalence of nosocomial infections has been associated to the growing problem of antimicrobial and detergent-resistant pathogens [8,9]. As such, proper use of effective disinfecting strategies in hospitals is necessary to abate this growing problem [10].

Ortho-phenyphenol is used as a fungicide and as an antibacterial agent in a wide variety of settings. OPP is used as a hospital disinfectant, and as a fungicide and disinfectant for wood preservation, the treatment of vegetables and citrus fruits and textile production [11,12]. Results from toxicological studies indicate that OPP administered in diet, leads to the formation of tumors in the urinary bladder of rats [13]. OPP has also been reported to cause sister-chromatid exchanges and chromosomal aberrations in Chinese hamster ovary cells (CHO-K1 cells) [14].

Despite the aforementioned detrimental effects of OPP and its many uses to combat microbial contamination, to our knowledge, the mechanism of action of OPP on bacterial pathogens has not been elucidated. Moreover, the use of OPP as a hospital disinfectant necessitates an understanding of the cellular functions that it affects in different pathogenic bacteria. This will facilitate the determination of its mode of action such that it can be more effectively utilized. Further, such information will expedite the development of efficient antimicrobials which target specific pathogenic bacteria and exert nominal effects on other species. In previous studies, whole genome microarrays have been successfully used to analyze the global transcriptomic response of *P. aeruginosa *to different antimicrobials. From these studies, specific cellular functions affected by the application of these antimicrobials were elucidated through the identification of signature genes that were up or downregulated [15-19].

To our knowledge, for the first time, we investigated the genome-wide changes in *P. aeruginosa *gene transcription upon exposure to 0.82 mM OPP for 20 and 60 minutes using Affymetrix *P. aeruginosa *GeneChip arrays. Our findings show that: (i) the transcription of genes encoding ribosomal, virulence and membrane proteins (including membrane transport systems) were upregulated after 20 and 60 minutes (ii) the transcription of genes that may allow transient switches to anaerobic respiration and swarming motility as stress responses were upregulated after 20 minutes (iii) after 60 minutes, amino acid anabolism and lipopolysaccharide synthesis were upregulated. (iv) after both 20 and 60 minutes of OPP treatment, the transcription of the genes encoding the ribosome modulation factor and the alternative sigma factor, RpoS were significantly downregulated.

## Results and discussion

### Growth inhibition of *P. aeruginosa *by OPP

**Figure 1:**
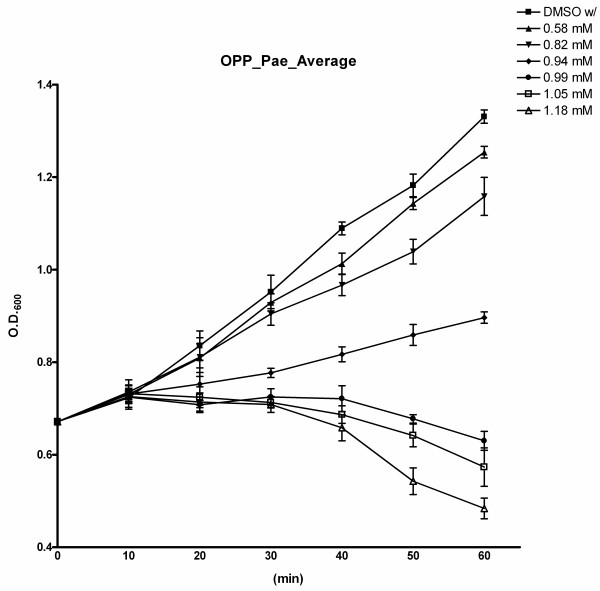
**Growth inhibition of *P. aeruginosa *treated with orthophenylphenol (OPP)**. Cell density was monitored as the OD_600 _in ten minute intervals. The OPP concentrations were as follows: 0 mM control with DMSO (filled square), 0.58 mM (filled triangle), 0.82 mM (inverted filled triangle), 0.94 mM (filled diamond), 0.99 mM (filled circle), 1.05 mM (empty square), 1.18 mM (empty triangle). Each data point was derived as the average of three separate experiments and the error bars represent the standard deviation obtained.

In order to determine a suitable sublethal concentration of OPP that will produce strong growth inhibition, *P. aeruginosa *was exposed to six concentrations of OPP dissolved in DMSO (0.58, 0.82, 0.94, 0.99, 1.05 and 1.18 mM), and growth inhibition was monitored at intervals of 10 minutes for 60 minutes. Note that the concentration of OPP that inhibits 90% of *P. aeruginosa *isolates (MIC_90_) has been reported to be 2000 mg/L (1.18 mM) [20]. In figure 1, the highest concentration of OPP used (1.18 mM) produced marked growth inhibition. Therefore, a lower sublethal concentration of 0.82 mM was selected as the test concentration since this concentration caused a non-drastic sublethal growth inhibition as seen in figure 1.

### Changes in the transcriptional profiles of *P. aeruginosa *in response to OPP

Four separate microarray experiments were performed in the absence (control) and in the presence (experimental) of 0.82 mM OPP. In order to investigate early and late changes in transcription in response to OPP, RNA was isolated after 20 and 60 minutes exposure to 0.82 mM OPP. To determine which genes showed significant changes in transcript level in response to OPP, the following criteria were applied: (i) the *p*-value for a Mann-Whitney test should be less than 0.05, (ii) an absolute fold change in transcript level should be equal to or greater than 2 (iii) a gene should have a present or marginal call (Affymetrix, Inc.) from 50% or more replicates on both experimental and control replicate sets. After a one-way ANOVA was performed, 1012 out of the 5900 genes that make up the *P. aeruginosa *genome were found to be statistically significant. Further analysis revealed that a total of 509 genes showed statistically marked upregulation (≥ 2-fold) or downregulation (≤ 2-fold) after 20 minutes and after 60 minutes exposure to OPP. The expression levels of the 5900 genes in the *P. aeruginosa *genome obtained from control experiments and after treatment with OPP (20 and 60 minutes) have been deposited in NCBI's gene Expression Omnibus [21] and can be accessed through the GEO series accession number: GSE10604 [22] (additional file 1).

### Functional classification of upregulated and downregulated genes

**Figure 2:**
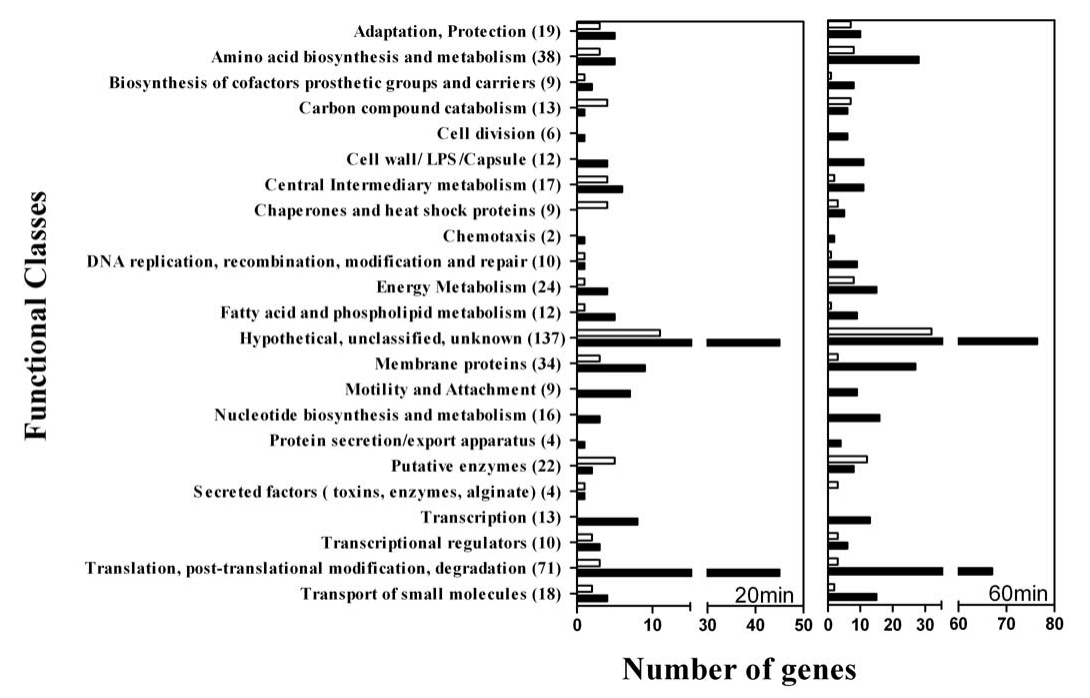
**Functional classification of statistically significant upregulated (filled bars) and downregulated (empty bars) genes after 20 minutes and 60 minutes exposure to 0.82 mM OPP**. The numbers in parentheses indicate the total number of genes for each functional class in both groups (a total of 509 genes).

In order to relate the up and downregulated genes to their functions, the 509 statistically significant genes were classified into different functional classes. Functional classes were obtained from the *P. aeruginosa *Community Annotation Project [23,24]. Figure 2 illustrates the grouping of up and down regulated genes at 20 and 60 minutes into different functional classes and the total number of genes in each class for the two treatment times. Note that a total of 137 genes were classified as "hypothetical, unclassified, unknown".

Figure 2 illustrates that in general at 60 minutes, there were more upregulated and downregulated genes in the functional classes, when compared to 20 minutes. In particular, genes belonging to the functional classes of "adaptation and protection", amino acid biosynthesis and metabolism", "biosynthesis of cofactors, prosthetic groups and carriers", "carbon compound catabolism", "cell division", "cell wall/LPS/capsule", "central intermediary metabolism", "chaperones and heat shock proteins", "DNA replication, recombination and repair", "energy metabolism", "fatty acid and phospholipid metabolism", "membrane proteins", "nucleotide biosynthesis and metabolism", "putative enzymes", "transcription", "transcriptional regulators", "translation, post-translational modification, degradation", and "transport of small molecules contained significantly more upregulated genes at 60 minutes.

Among the downregulated genes, the functional classes of: "adaptation and protection", "amino acid biosynthesis and metabolism", "carbon compound catabolism" and "energy metabolism" contained significantly more downregulated genes at 60 minutes compared to 20 minutes. The marked differences between the numbers of upregulated and downregulated genes after 20 minutes exposure compared to 60 minutes of treatment may be related to the growth inhibition observed following exposure to OPP.

### Grouping of functionally classified up and down regulated genes

**Figure 3:**
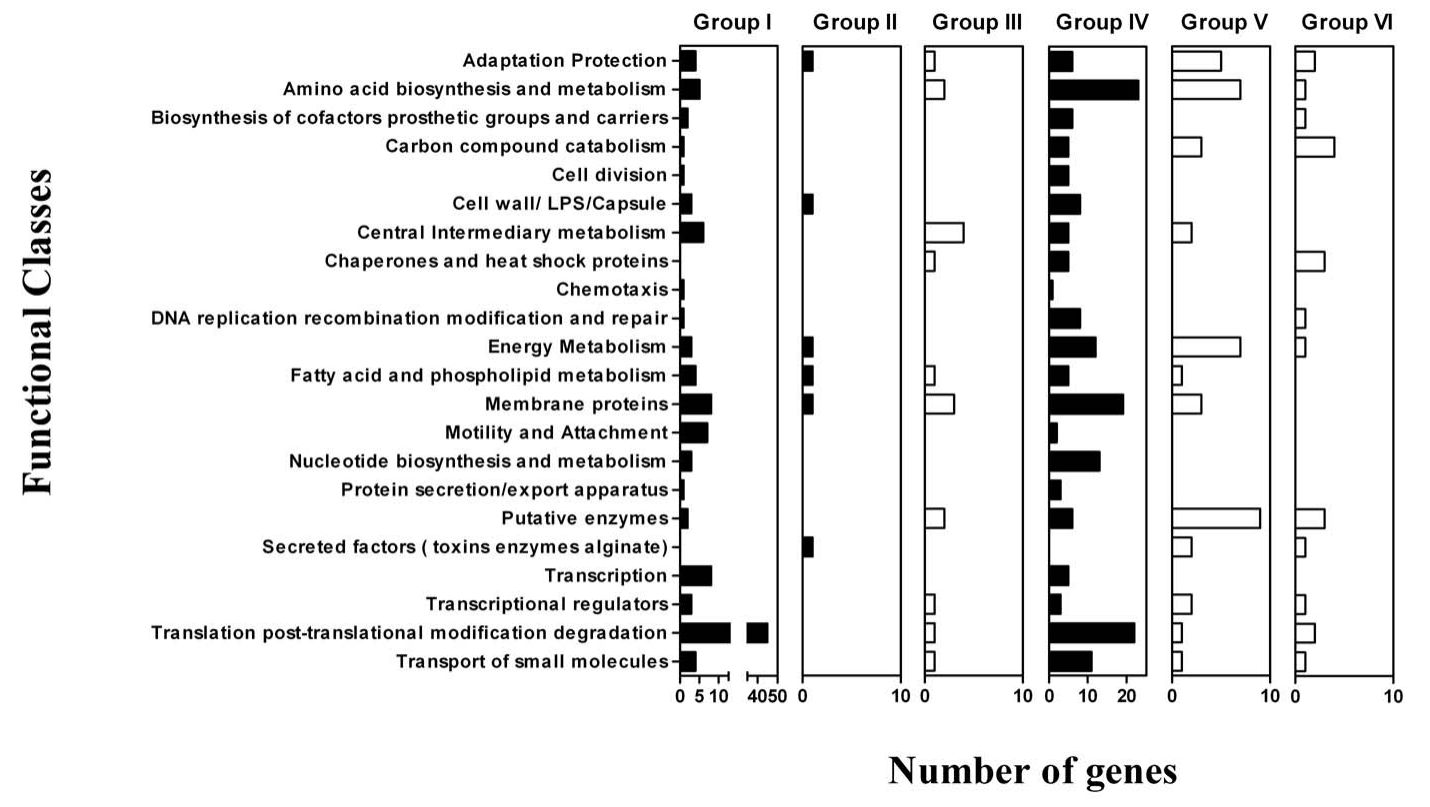
**Classification of differentially regulated 372 genes into six groups based on their transcription directions after 20 and 60 minutes exposure to OPP**. Note that genes belonging to the functional class "hypothetical, unclassified, unknown" (137 genes) are not represented in this figure. Filled bars indicate upregulation either after one or both treatment times. Empty bars indicate downregulation either after one or both treatment times. Group I is made up of genes upregulated after both exposure times. Group II contains genes upregulated at 20 minutes, with no significant changes after 60 minute exposure. Group III consists of genes downregulated after 20 minutes, with no significant changes upon 60 minutes of treatment. Group IV is made up of genes that were upregulated in response to 60 minutes of treatment. Group V is made up of genes that were downregulated upon 60 minutes of treatment. Group VI is made up of genes that were downregulated upon both exposure times.

To further analyze the 509 upregulated and downregulated genes, we removed the 137 genes belonging to the class designated as "hypothetical, unclassified, unknown". The remaining 372 genes were placed in six groups based on their transcription directions. Figure 3 illustrates the six different groups and the total number of genes in each group. Group I contains genes that were upregulated after 20 and 60 minutes. Group II is made up of genes that were upregulated after 20 minutes only. Group III contains genes that were downregulated upon 20 minutes of exposure to OPP. Group IV contains genes that were upregulated after 60 minutes only. Group V is made up of genes that were downregulated only after 60 minutes exposure to OPP. Group VI contains genes that were downregulated after both 20 and 60 minutes exposure to OPP. All of the genes discussed in this report can be found in additional file 2. However, for clarity and to facilitate the reading of this report, the genes discussed below in the six groups are indicated in table 1.

**Table 1:** List of significantly up or downregulated *P. aeruginosa *genes that are discussed in this report

**Affymetrix ORF #**	**Probe ID**	**^a^20 minutes**		**^a^60 minutes**		**Description**	**Symbol**	**Functional class**
		**^b^Fold change**	**P value**	**^b^Fold change**	**P value**			

**Group I: Upregulation (20 min) – Upregulation (60 min)**								
PA1964_at	PA1964	2.029	0.0112	2.381	0.0112	probable ATP-binding component of ABC transporter		Transport of small molecules
PA2760_at	PA2760	2.152	0.0416	2.94	0.0416	probable outer membrane protein precursor		Transport of small molecules
PA4687_hitA_at	PA4687	2.219	0.00808	2.069	0.00808	ferric iron-binding periplasmic protein HitA	*hit*A	Transport of small molecules
PA2743_infC_at	PA2743	2.044	0.000327	2.634	0.000327	translation initiation factor IF-3	*inf*C	Translation, post-translational modification, degradation
PA2619_infA_at	PA2619	2.14	0.000526	3.644	0.000526	translation initiation factor	*inf*A	Translation, post-translational modification, degradation
PA4266_fusA1_at	PA4266	2.18	0.012	3.88	0.012	elongation factor G	*fus*A1	Translation, post-translational modification, degradation
PA3655_tsf_at	PA3655	2.197	0.017	5.495	0.017	elongation factor Ts	*tsf*	Translation, post-translational modification, degradation
PA4665_prfA_at	PA4665	2.426	0.00398	2.774	0.00398	peptide chain release factor 1	*prf*A	Translation, post-translational modification, degradation
PA4273_rplA_at	PA4273	2.725	0.0349	6.018	0.0349	50S ribosomal protein L1	*rpl*A	Translation, post-translational modification, degradation
PA3656_rpsB_at	PA3656	2.543	0.00953	7.267	0.00953	30S ribosomal protein S2	*rps*B	Translation, post-translational modification, degradation
PA4744_infB_at	PA4744	2.783	0.0134	3.826	0.0134	translation initiation factor IF-2	*inf*B	Translation, post-translational modification, degradation
PA4934_rpsR_at	PA4934	2.894	0.000276	6.619	0.000276	30S ribosomal protein S18	*rps*R	Translation, post-translational modification, degradation
PA4255_rpmC_at	PA4255	2.927	0.00331	6.655	0.00331	50S ribosomal protein L29	*rpm*C	Translation, post-translational modification, degradation
PA4528_pilD_at	PA4528	2.144	0.014	2.65	0.014	type 4 prepilin peptidase PilD	*pil*D	Motility & Attachment
PA0408_pilG_at	PA0408	2.294	0.0144	4.026	0.0144	twitching motility protein PilG	*pil*G	Chemotaxis
PA5041_pilP_at	PA5041	2.169	0.00817	2.232	0.00817	type 4 fimbrial biogenesis protein PilP	*pil*P	Motility & Attachment
PA0410_pilI_at	PA0024	2.188	0.0484	2.267	0.0484	twitching motility protein PilI	*pil*I	Motility & Attachment
PA5042_pilO_at	PA5042	2.26	0.000345	2.056	0.000345	type 4 fimbrial biogenesis protein PilO	*pil*O	Motility & Attachment
PA4527_pilC_at	PA4527	2.27	0.00205	2.678	0.00205	still frameshift type 4 fimbrial biogenesis protein PilC	*pil*C	Motility & Attachment
PA5043_pilN_at	PA5043	2.34	0.000628	2.914	0.000628	type 4 fimbrial biogenesis protein PilN	*pil*N	Motility & Attachment
PA5044_pilM_at	PA5044	2.893	0.00702	3.12	0.00702	type 4 fimbrial biogenesis protein PilM	*pil*M	Motility & Attachment
PA4688_hitB_at	PA4688	2.282	0.0261	2.513	0.0261	iron (III)-transport system permease HitB	*hit*B	Membrane proteins
PA4747_secG_at	PA4747	2.294	0.00628	3.795	0.00628	secretion protein SecG	*sec*G	Membrane proteins
PA4243_secY_at	PA4243	2.914	9.26E-05	6.799	9.26E-05	secretion protein SecY	*sec*Y	Membrane proteins
PA4276_secE_at	PA4276	2.274	2.07E-05	4.198	2.07E-05	secretion protein SecE	*sec*E	Protein secretion/export apparatus
PA2968_fabD_at	PA2968	2.137	0.0131	3.506	0.0131	malonyl-CoA-[acyl-carrier-protein] transacylase	*fab*D	Fatty acid and phospholipid metabolism
PA1609_fabB_at	PA1609	2.373	0.0147	2.855	0.0147	beta-ketoacyl-ACP synthase I	*fab*B	Fatty acid and phospholipid metabolism
PA2967_fabG_at	PA2967	2.387	0.00109	3.416	0.00109	3-oxoacyl-[acyl-carrier-protein] reductase	*fab*G	Fatty acid and phospholipid metabolism
PA1610_fabA_at	PA1610	2.864	0.00295	4.071	0.00295	beta-hydroxydecanoyl-ACP dehydrase	*fab*A	Fatty acid and phospholipid metabolism
PA3645_fabZ_at	PA3645	2.854	2.25E-05	4.671	2.25E-05	(3R)-hydroxymyristoyl-[acyl carrier protein] dehydratase	*fab*Z	Cell wall/LPS/capsule
PA5556_atpA_at	PA5556	2.202	0.00136	3.907	0.00136	ATP synthase alpha chain	*atp*A	Energy metabolism
PA5491_at	PA5491	2.354	0.0049	2.854	0.0049	probable cytochrome		Energy metabolism
PA5561_atpI_at	PA5561	2.558	0.0015	2.542	0.0015	ATP synthase protein I	*atp*I	Energy metabolism
PA3818_at	PA4263	2.746	0.00537	5.447	0.00537	extragenic suppressor protein SuhB	*Suh*B	Adaptation, protection
PA4743_rbfA_at	PA4743	2.824	0.0177	4.063	0.0177	ribosome-binding factor A	*rbf*A	Adaptation, protection
PA5117_typA_at	PA5117	3.136	0.000343	5.723	0.000343	regulatory protein TypA	*Typ*A	Adaptation, protection
**Group II: Upregulation (20 min) – No change (60 min)**								
PA0524_norB_at	PA0524	3.869	0.0456			nitric-oxide reductase subunit B	*nor*B	Energy metabolism
PA3479_rhlA_at	PA3479	2.376	0.0364			rhamnosyltransferase chain A	*rhl*A	Secreted Factors (toxins, enzymes, alginate)
PA0177_at	PA0177	2.683	0.00294			probable purine-binding chemotaxis protein		Adaptation, protection
**Group III: Downregulation (20 min) – No change (60 min)**								
PA2193_hcnA_at	PA2193	-2.268	0.0324			hydrogen cyanide synthase HcnA	*hcn*A	Central intermediary metabolism
PA2194_hcnB_at	PA2194	-2.762	0.018			hydrogen cyanide synthase HcnB	*hcn*B	Central intermediary metabolism
PA2195_hcnC_at	PA2195	-2.183	0.0437			hydrogen cyanide synthase HcnC	*hcn*C	Central intermediary metabolism
PA4385_groEL_at	PA4385	-2.16	0.00513			GroEL protein	*gro*EL	Chaperones & heat shock proteins
PA4542_clpB_at	PA4542	-2.77	0.00253			ClpB protein	*clp*B	Translation, post-translational modification, degradation
**Group IV: No change(20 min) – Upregulation(60 min)**								
PA4272_rplJ_at	PA4272			5.918	0.00886	50S ribosomal protein L10	*rpl*J	Translation, post-translational modification, degradation
PA4271_rplL_at	PA4271			5.898	0.00319	50S ribosomal protein L17/L12	*rpl*L	Translation, post-translational modification, degradation
PA2740_pheS_at	PA2740			3.323	0.000548	phenylalanyl-tRNA synthetase, alpha-subunit	*phe*S	Translation, post-translational modification, degradation
PA4240_rpsK_at	PA4240			3.283	0.0292	30S ribosomal protein S11	*rps*K	Translation, post-translational modification, degradation
PA4267_rpsG_at	PA4267			3.104	0.0138	30S ribosomal protein S7	*rps*G	Translation, post-translational modification, degradation
PA2739_pheT_at	PA2739			2.142	0.0375	phenylalanyl-tRNA synthetase, beta subunit	*phe*T	Translation, post-translational modification, degradation
PA3987_leuS_at	PA3987			2.632	0.0216	leucyl-tRNA synthetase	*leu*S	Amino acid biosynthesis and metabolism
PA0009_glyQ_at	PA0009			2.568	0.000361	glycyl-tRNA synthetase alpha chain	*gly*Q	Amino acid biosynthesis and metabolism
PA3525_argG_at	PA3525			2.45	0.0411	argininosuccinate synthase	*arg*G	Amino acid biosynthesis and metabolism
PA3167_serC_at	PA3167			2.345	0.0396	3-phosphoserine aminotransferase	*ser*C	Amino acid biosynthesis and metabolism
PA0904_lysC_at	PA0904			2.337	0.00528	aspartate kinase alpha and beta chain	*lys*C	Amino acid biosynthesis and metabolism
PA5263_argH_at	PA5263			2.29	0.00853	argininosuccinate lyase	*arg*H	Amino acid biosynthesis and metabolism
PA4007_proA_at	PA4007			2.282	0.0231	gamma-glutamyl phosphate reductase	*pro*A	Amino acid biosynthesis and metabolism
PA5277_lysA_at	PA5277			2.247	0.0334	diaminopimelate decarboxylase	*lys*A	Amino acid biosynthesis and metabolism
PA5143_hisB_at	PA5143			2.246	0.00461	imidazoleglycerol-phosphate dehydratase	*his*B	Amino acid biosynthesis and metabolism
PA5039_aroK_at	PA5039			2.234	0.013	shikimate kinase	*aro*K	Amino acid biosynthesis and metabolism
PA0018_fmt_at	PA0018			2.222	0.0137	methionyl-tRNA formyltransferase	*fmt*	Amino acid biosynthesis and metabolism
PA5067_hisE_at	PA5067			2.212	0.00214	phosphoribosyl-ATP pyrophosphohydrolase	*his*E	Amino acid biosynthesis and metabolism
PA3482_metG_at	PA3482			2.043	0.0312	methionyl-tRNA synthetase	*met*G	Amino acid biosynthesis and metabolism
PA5119_glnA_at	PA5119			2.038	0.0442	glutamine synthetase	*gln*A	Amino acid biosynthesis and metabolism
PA4439_trpS_at	PA4439			2.002	0.0155	tryptophanyl-tRNA synthetase	*trp*S	Amino acid biosynthesis and metabolism
PA0903_alaS_at	PA0903			2.099	0.0102	alanyl-tRNA synthetase	*ala*S	Transcription, RNA processing and degradation
PA4602_glyA3_at	PA4602			3.347	2.83E-05	serine hydroxymethyltransferase	*gly*A3	Amino acid biosynthesis and metabolism
PA0971_tolA_at	PA0971			2.249	0.000381	TolA protein	*tol*A	Transport of small molecules
PA5479_gltP_at	PA5479			2.413	0.00978	proton-glutamate symporter	*glt*P	Membrane proteins
PA3821_secD_at	PA3821			3.516	0.00324	secretion protein SecD	*sec*D	Membrane proteins
PA3820_secF_at	PA3820			2.206	0.0411	secretion protein sec F Protein secretion	*sec*F	Protein secretion/export apparatus
PA5070_tatC_at	PA5070			2.312	0.0209	transport protein TatC	*tat*C	Membrane proteins
PA0973_oprL_at	PA0973			2.281	0.00672	Peptidoglycan associated lipoprotein OprL precursor	*opr*L	Membrane proteins
PA0280_cysA_at	PA0280			2.018	0.0139	sulfate transport protein CysA	*cys*A	Transport of small molecules
PA5217_at	PA5217			2.141	0.00709	probable binding protein component of ABC iron transporter		Transport of small molecules
PA0295_at	PA0295			2.643	0.0147	probable periplasmic polyamine binding protein		Transport of small molecules
PA4461_at	PA4461			2.24	0.00116	probable ATP-binding component of ABC transporter		Transport of small molecules
PA5503_at	PA5503			2.229	0.000446	probable ATP-binding component of ABC transporter		Transport of small molecules
PA4450_murA_at	PA4450			2.855	0.00044	UDP-N-acetylglucosamine 1-carboxyvinyltransferase	*mur*A	Cell wall/LPS/capsule
PA3644_lpxA_at	PA3644			2.83	0.00038	UDP-N-acetylglucosamine acyltransferase	*lpx*A	Cell wall/LPS/capsule
PA5276_lppL_i_at	PA5276			2.786	0.0149	Lipopeptide LppL precursor	*lpp*L	Cell wall/LPS/capsule
PA3643_lpxB_at	PA3643			2.561	0.0247	lipid A-disaccharide synthase	*lpx*B	Cell wall/LPS/capsule
PA5012_waaF_at	PA5012			2.092	0.00978	heptosyltransferase II	*waa*F	Cell wall/LPS/capsule
PA3337_rfaD_at	PA3337			3.397	0.0498	ADP-L-glycero-D-mannoheptose 6-epimerase	*rfa*D	Cell wall/LPS/capsule
PA5129_grx_at	PA5129			3.855	0.0334	glutaredoxin	*grx*	Energy metabolism
PA5555_atpG_at	PA5555			3.527	0.0287	ATP synthase gamma chain	*atp*G	Energy metabolism
PA5554_atpD_at	PA5554			3.011	0.00384	ATP synthase beta chain	*atp*D	Energy metabolism
PA3621_fdxA_at	PA3621			3.01	0.0042	ferredoxin I	*fdx*A	Energy metabolism
PA5559_atpE_at	PA5559			2.734	0.0205	atp synthase C chain	*atp*E	Energy metabolism
PA5560_atpB_at	PA5560			2.433	0.0148	ATP synthase A chain	*atp*B	Energy metabolism
PA2995_nqrE_at	PA2995			2.216	0.00617	Na+-translocating NADH:quinone oxidoreductase subunit Nqr5	*nqr*E	Energy metabolism
PA5553_atpC_at	PA5553			2.175	0.00502	ATP synthase epsilon chain	*atp*C	Energy metabolism
PA0362_fdx1_at	PA0362			2.125	0.000346	ferredoxin [4Fe-4S]	*fdx*1	Energy metabolism
PA3527_pyrC_at	PA3527			2.218	0.0211	dihydroorotase	*pyr*C	Nucleotide biosynthesis and metabolism
PA5331_pyrE_at	PA5331			2.156	0.0166	orotate phosphoribosyltransferase	*pyr*E	Nucleotide biosynthesis and metabolism
PA3654_pyrH_at	PA3654			2.434	0.0212	uridylate kinase	*pyr*H	Nucleotide biosynthesis and metabolism
PA3763_purL_at	PA3763			2.279	0.00792	phosphoribosylformylglycinamidine synthase	*pur*L	Nucleotide biosynthesis and metabolism
**Group V: No change(20 min) – Downregulation(60 min)**								
PA1174_napA_at	PA1174			-3.425	0.0251	periplasmic nitrate reductase protein NapA	*nap*A	Energy metabolism
PA1175_napD_at	PA1175			-2.949	0.0134	NapD protein of periplasmic nitrate reductase	*nap*D	Energy metabolism
PA1176_napF_at	PA1176			-2.949	0.024	ferredoxin protein NapF	*nap*F	Energy metabolism
PA1173_napB_at	PA1173			-3.077	0.00344	cytochrome c-type protein NapB precursor	*nap*B	Energy metabolism
PA2248_bkdA2_at	PA2248			-5.076	0.0072	2-oxoisovalerate dehydrogenase (beta subunit)	*bkd*A2	Amino acid biosynthesis and metabolism
PA2247_bkdA1_at	PA2247			-9.434	0.00376	2-oxoisovalerate dehydrogenase (alpha subunit)	*bkd*A1	Amino acid biosynthesis and metabolism
PA2250_lpdV_at	PA2250			-3.497	0.00576	lipoamide dehydrogenase-Val	*lpd*V	Amino acid biosynthesis and metabolism
**Group VI: Downregulation (20 min) – Downregulation (60 min)**								
PA3049_rmf_at	PA3049	-6.25	0.000723	-25.907	0.000723	ribosome modulation factor	*rmf*	Translation, post-translational modification, degradation
PA3622_rpoS_at	PA3622	-2.653	0.00961	-2.967	0.00961	sigma factor RpoS	*rpo*S	Transcriptional regulators
PA4762_grpE_at	PA4762	-2.915	0.00501	-0.479	0.00501	heat shock protein GrpE	*grp*E	DNA replication, recombination, modification and repair
PA5054_hslU_at	PA5054	-3.226	0.000316	-0.428	0.000316	heat shock protein HslU	*hsl*U	Chaperones & heat shock proteins
PA5053_hslV_at	PA5053	-2.597	0.00113	-0.352	0.00113	heat shock protein HslV	*hsl*V	Chaperones & heat shock proteins
PA1596_htpG_at	PA1596	-2.695	0.00706	-0.434	0.00706	heat shock protein HtpG	*htp*G	Chaperones & heat shock proteins

Genes are categorized by their transcription patterns and related functions. The microarray results are the mean of four replicates of each gene.

^a^The microarray results are the mean of four replicates of each gene.

^b^The fold change is a positive number when the expression level in the experiment increased compared to the control and is a negative number when the expression level in the experiment decreased.

#### Group I: genes upregulated at 20 and 60 minutes exposure

Group 1 consisted of genes that were induced both at 20 and 60 minutes exposure to OPP (additional file 2). The most distinctive functional class in this group was "translation, post-translational modification and degradation" which contained 45 genes (additional file 2). This functional class contained several 30 and 50S ribosomal proteins. The two most upregulated 30 and 50S ribosomal proteins are indicated on table 1. A complete list of ribosomal proteins in this group can be found in additional file 2. Group 1 also contained genes coding for translation initiation factors: PA2619, PA4744 and PA 2743 (*inf*A, *inf*B and *inf*C), elongation factors G and Ts: PA4266, PA3655 (*fus*A1, *tsf*) and peptide chain release factor: (*prf*A) PA4665 (table 1). The upregulation of these genes after both 20 and 60 minutes suggests that protein synthesis is affected in *P. aeruginosa *upon exposure to OPP and even after prolonged exposure (60 minutes). This may reflect a cellular protective response, whereby proteins involved in stress response are synthesized. The upregulation of the cold shock protein: PA4743 (*rbf*A) and the heat shock protein: PA4263 (*suh*B) which are involved in stress response supports this hypothesis. The observed upregulation of translation may also indicate an increase in the synthesis of virulence factors, which can be produced in response to environmental stress. Previous studies have suggested that the pathogenesis of *Staphylococcus aureus *is stimulated as a protective response against antimicrobial treatments [25,26]. In line with this hypothesis was the upregulation of PA5117 (*typ*A), which has been suggested to be relevant for pathogenesis in *Escherichia coli *when it is tyrosine phosphorylated [27].

Among the genes in the functional class of "membrane proteins" in this group, we observed three proteins of the SecY system: PA4243 (*sec*Y), PA4276 (*sec*E) and PA4747 *sec*G) were upregulated. In gram negative bacteria, the Sec system is utilized for the secretion of degradative enzymes, virulence factors, toxins and proteins across the cytoplasmic membrane and for the insertion of proteins into the cytoplasmic membrane [28], allowing for growth and survival. The concomitant upregulation of genes involved in the Sec system and genes involved in translation is possibly indicative of the transport of synthesized proteins across the cell membrane.

Concurrent with the induction of genes of the Sec system was the upregulation of genes involved in membrane associated transport of small molecules notably, PA4687: ferric iron-binding periplasmic protein (*hit*A), PA1964: probabale ATP-binding component of ABC transporter, PA2760: probable outer membrane protein precursor and PA4688: Iron III-transport system permease (*hit*B). These results are in agreement with those of a recent study that demonstrated that the *hit*A and *hit*B genes were 2- to 8-fold upregulated in *P. aeruginosa *in response to a two hour exposure to 10 mM hydrogen peroxide [17]. HitA, HitB and HitC (a nucleotide binding protein) encoded by the *hit*ABC operon facilitate iron acquisition from the periplasm [29]. In contrast, a previous study indicated that the *hit*AB genes were downregulated approximately 7- and 6- fold in *P. aeruginosa *treated with sodium hypochlorite for 20 minutes [18]. Therefore the upregulation of *hit*A (ferric iron-binding periplasmic protein) and *hit*B (Iron III-transport system permease) in this study is suggestive of active iron uptake, which is essential for bacterial growth and an important determinant of bacterial virulence [29].

It is worth noting that eight type IV pilus assembly proteins belonged to this group including *pil *C, D, G, I, M, N, O and P. Type IV pili have been implicated in the pathogenicity of gram negative bacteria, and mediate cellular functions such as twitching motility, host-cell adhesion and cell signaling [30]. The expression of type IV pili is necessary for colonization and maturation of *P. aeruginosa *biofilms on a variety of surfaces [31]. Considering that the cellular functions noted above generally mediate virulence and cell survival, it is therefore possible that the upregulation of these genes may be associated with protection and concomitant survival of *P. aeruginosa *when treated with OPP.

Five genes involved in fatty acid biosynthesis (*fab*A, B, D, G and Z) were also categorized under group I. A previous study that investigated the proteomic response of *P. putida *to phenol-induced stress noted that several enzymes involved in fatty acid biosynthesis, including FabB and FabH2 were upregulated approximately 2 fold and 4 fold respectively when treated with phenol [32]. Although FabA was not mapped in the aforementioned study, it was suggested that under phenol stress, the expression level of FabA correlated with the upregulation of FabB. Our study corroborates this theory, as both *fab*A and *fab*B were upregulated approximately 3 and 4 fold respectively following 60 minutes exposure to OPP (t). In contrast, *fab*H2 was downregulated in *P. aeruginosa *after 20 minutes of treatment with peracetic acid [16].

With respect to energy metabolism, the genes: PA5561 (*atp*1), PA2354, (probable cytochrome) and PA5556 (ATP synthase alpha chain), which are involved in ATP synthesis associated with oxidative phosphorylation and the electron transport chain were upregulated at both 20 and 60 minutes. This result suggests that oxidative phosphorylation is possibly a major route for energy production in *P. aeruginosa *treated with OPP.

#### Group II: genes upregulated upon 20 minutes exposure

Group II contained 13 genes, the least number of genes among the six groups (additional file 2). The most upregulated gene in this group was *nor*B (nitric oxide reductase subunit B), with an approximately four fold increase in transcription (table 1). The nitric oxide reductase enzyme is a membrane bound cytochrome *bc *complex which has been reported to be expressed under anaerobic conditions in *P. stutzeri *[33-35]. NorB is the catalytic component of the NorBC complex and harbors low and high spin and low spin ferric heme proteins [36]. Recent experimental evidence suggests that coupled with electron transfer, proton uptake by NorB occurs from the periplasmic side of the bacterial cell membrane [33]. As such, nitrate can be used as the terminal electron acceptor instead of oxygen under anaerobic conditions, with nitric oxide being one of the intermediates in the reduction of nitrate to dinitrogen in the denitrification process [33]. Nitric oxide produced during denitrification is highly toxic to the cell and relies on the scavenging activity of nitric oxide reductase for cell survival [34]. A previous study [37] demonstrated that several genes involved in anaerobic respiration were upregulated in *S. aureus *after 20 minutes of exposure to peracetic acid, suggesting the possibility of a shift to anaerobic respiration in response to oxidative stress.

Another gene of interest in this group was PA3479: rhamnosyl transferase chain A (*rhl*A). The *rhl*AB operon catalyzes the first gylcosyl transfer reaction required for the synthesis of rhamnolipids [38,39]. Rhamnolipids have been found in high levels in the sputum of cystic fribrosis patients and are classified as virulence factors [40]. Rhamnolipids have been postulated to act as biosurfactants that facilitate surface colonization [41]. The multicellular nature of both biofilms and cells undergoing swarming motility indicate that both phenomena are related [42,43]. Rhamnosyl transferase chain A has been found to be critical for the exhibition of swarming motility by *P. aeruginosa*, which is important for environmental adaptation [44,45]. It has also been demonstrated that *P. aeruginosa *mutants lacking type IV pili and flagella are unable to swarm [45]. The upregulation of type IV pili assembly genes at 20 and 60 minutes (group I) supports the possibility that *P. aeruginosa *treated with OPP may exhibit swarming motility as a stress response. The finding that the *rhl*A gene exhibited no change in its expression level at 60 minutes also supports this hypothesis. Moreover, the probable purine binding chemotaxis protein, PA0177, which belongs to the group of flagellar assembly proteins, was also upregulated in this group.

Previous studies have revealed that low iron levels significantly stimulated swarming motility, thereby preventing biofilm formation [44,46]. From this observation, it was hypothesized that in an unfavorable nutritional environment, biosurfactant production and surface motility are over expressed in order to prevent *P. aeruginosa *from settling into a biofilm [44]. Our results are in line with this hypothesis, considering that the ferric iron-binding periplasmic protein and the iron III-transport system permease (*hit*A and *hit*B) were upregulated after both 20 and 60 minutes (group I) of exposure to OPP, suggesting active iron uptake.

#### Group III: genes downregulated upon 20 minutes exposure

One of the characteristics of this group was the downregulation of genes involved in hydrogen cyanide production: PA2193 (*hcn*A), PA2194 (*hcn*B), PA2195 (*hcn*C). The *hcn*ABC genes eoncode a cyanide synthase, which forms hydrogen cyanide from glycine [47]. These results are similar to those of a previous study where the *hcn*A and B genes were downregulated in *P. aeruginosa *treated with peracetic acid for 20 minutes [16]. Hydrogen cyanide is considered an extracellular virulence factor of *P*. *aeruginosa *and its production is transcriptionally regulated by the anaerobic regulator ANR and the quorum-sensing regulators LasR and PhlR [48]. It has been established that hydrogen cyanide is optimally produced when cell densities are high during the transition from exponential to stationary phase [49]. *P. aeruginosa *does not produce cyanide when it is grown under anaerobic conditions, with nitrate being used as the terminal electron acceptor [50]. The down regulation of genes responsible for cyanide production, therefore, supports the possibility that *P. aeruginosa *experiences an oxygen limiting state characterized by a transient switch to anaerobic metabolism. The upregulation of the nitric oxide reductase enzyme (*nor*B) in group II supports this theory. Further, the *hcn*ABC genes did not exhibit a change in expression levels at 60 minutes, indicating resumption of aerobic metabolism.

Also in this group was the *gro*EL gene which encodes a heat shock protein. In a previous study, the expression of *gro*EL was unchanged in *P. putida *exposed to phenol [32]. The *clp*B gene which encodes an ATP dependent protease that functions as part of a chaperone network necessary for the recovery of stress induced protein aggregates was downregulated 2.7 fold. In contrast, the *clp*B gene has been shown to be upregulated 2.4 fold in *P. putida *treated with phenol [32].

#### Group IV: genes upregulated upon 60 minutes exposure

Group IV consists of genes whose expression levels increased only in response to 60 minutes of exposure to OPP (additional file 2). This group contained the highest number of genes (227 genes) among the six groups generated based on gene transcription direction. The significantly higher number of genes in this group compared with the number of genes in group II (genes upregulated upon 20 minutes exposure), suggests that *P. aeruginosa *significantly adjusts its transcriptional profile after 60 minutes of treatment with OPP.

The most dominant classes in this group were "amino acid biosynthesis and metabolism", "membrane proteins", "nucleotide biosynthesis and metabolism", "translation, post-translational modification and degradation" and "transport of small molecules".

Compared to the other functional classes in this group, a higher number of genes were involved in amino acid biosynthesis and in translation, post-translational modification and degradation. Active protein synthesis was reflected in the upregulation of several genes coding for 30 and 50S ribosomal proteins, aminoacyl-tRNA synthetases associated with alanine (*ala*S), formylmethionine (*fmt*), glycine (*gly*Q), leucine (*leu*S), methionine and selenomethionine (*met*G), phenylalanine (*phe*S and *phe*T) and tryptophan (*trp*S). In addition, genes involved in the biosynthesis of several amino acids: arginine and proline (*arg*G, *arg*H), glutamine (*gln*A), lysine (*lys*A, *lys*C), glutamate (*pro*A, *glt*P), histidine (*his*B, *his*E), phenylalanine (*aro*K), serine (*ser*C, *glyA*3), were also upregulated. These results are similar to those of a previous study [32] which indicated that the following enzymes were induced under phenol stress in *P. putida*: ArgD, ProA, GltD (involved in the glutamate biosynthetic pathway), TrpB and TrpS (belonging to aromatic amino acid biosynthetic pathways), and CysK and GlyA-2 (involved in the serine biosynthetic pathway). Based on these results, the authors suggested that phenol-stressed cells may experience amino acid deficits, and hence a shortage of proteins required for growth and survival [32]. Interestingly it has been shown that *E. coli *can adjust its rate of tryptophan biosynthesis following a shift to stressful nutritional conditions [51]. In contrast, a similar study in our laboratory investigating the effect of OPP on *S. aureus *[84] revealed that several genes involved in amino acid anabolism and specifically lysine and diaminopimelic acid (DAP) biosynthesis were markedly downregulated. This suggests that the effect of 0.82 mM OPP on amino acid metabolism in *P. aeruginosa *and *S. aureus *differ.

Among the genes coding for proteins involved in the transport of small molecules was the *tol*A gene, which codes for the TolA protein. The TolA protein is an inner membrane protein belonging to the TolQRAB protein complex [52] and is necessary for the uptake of the group A colicins and Tol-dependent phage [53,54]. Tol proteins are also required to maintain the integrity of the bacterial cell envelope structure [52,55]. Mutations in TolA have been shown to cause increased sensitivity to detergents and certain antimicrobials and the leakage of periplasmic proteins [56]. The upregulation of *tol*A after 60 minutes of exposure to OPP suggests a protective role for TolA, possibly related to the maintenance of the cell membrane structure.

In line with the upregulation of genes in the class of "transport of small molecules" after 60 minutes, was the induction of several genes belonging to the classes of "membrane proteins" and "protein secretion/export apparatus". The upregulation of the glycerol-3-phosphate transporter gene (*glt*P) and the proton glutamate symporter (*glt*P) is indicative of active transport across the cell membrane. Further evidence of translocation was seen in the upregulation of the components of translocation pathways such as the Sec dependent pathway (*sec*D, *sec*F) which is driven by ATP hydrolysis and the twin-arginine translocation (Tat) pathway (*tat*C) which uses energy derived from the proton motive force to translocate proteins across the cytoplasmic membrane [57,58]. Interestingly, *E. coli *with mutations in *tat*C, which is critical for the functioning of the Tat system, show pleitropic defects in the cell envelope, leading to hypersensitivity to some detergents and drugs [59,60]. The peptidoglycan associated lipoprotein precursor (*opr*L), which has been reported to play a protective role against hydrogen peroxide treatment in biofilms of *P. aeruginosa *[61] was also upregulated. Further, several genes encoding proteins in the ABC transport system, including the sulfate transport protein (*cys*A), probable protein binding component of iron ABC transporter (PA5217), probable periplasmic polyamine binding protein (PA0295) and probable ATP binding component of ABC transporters (PA4461, PA5503) were also upregulated in this group. These findings possibly imply that membrane components of *P. aeruginosa *were altered and that activated and or facilitated transport of ions, sugars, amino acids and other solutes necessary for cell survival was boosted after 60 minutes of exposure to OPP. It therefore appears that both the maintenance of active transport across and the integrity of the cell membrane are necessary for cell survival after 60 minutes of OPP treatment.

This group also contained six genes: *rfa*D, *mur*A, *lpx*A, *lpp*L, *lpx*B and *waa*F that are involved in the lipopolysaccharide (LPS) biosynthetic pathway. LPS is the main component of the outer cell wall and upregulation of its synthesis suggests that adaptation to OPP treatment in *P. aeruginosa *may involve cell wall modification. Similar results were obtained in *P. putida*, where the LpxC, MurA and the VacJ (a putative lipoprotein) proteins were upregulated after exposure to phenol [32].

Another predominant functional group in this class contained genes involved in energy metabolism. Genes encoding several components of the F_1_ATP synthase (*atp*B, *atp*C, *atp*D, *atp*G) involved ATP generation by oxidative phosphorylation and elements mediating electron transfer (glutaredoxin (PA5129), ferredoxin I (PA3621), ferredoxin (PA0362) and the Na^+^-translocating NADH: quinone oxidoreductase subunit Nqr5 (PA2995) were upregulated. Other components involved in the oxidative phosphorylation pathway: (PA5561 (*atp*1) and PA5556 (ATP synthase alpha chain) were upregulated after both 20 and 60 minutes (PA5561 (*atp*1) and PA5556 (ATP synthase alpha chain). This corroborates the theory that energy production through this route is essential for OPP-treated cells.

An interesting observation was the upregulation of several genes involved in the biosynthesis of purines and pyrimidines after 60 minutes of treatment with OPP. The genes: *pyr*C, *pyr*E, *pyr*H, belonging to the pyrimidine biosynthetic operon that has been described in *Bacillus subtilis *[62] and the *pur*L gene involved in purine biosynthesis [63] were upregulated, suggesting that an increase in nucleotide biosynthesis may contribute to the adaptive response of *P. aeruginosa *to OPP. In contrast, the quantities of the PurM, PurL and PyrH proteins have been reported to be downregulated after exposure to phenol for 60 minutes [32].

#### Group V: genes downregulated upon 60 minutes exposure

This group contained a total of 70 genes (additional file 2) that were downregulated after 60 minutes of treatment with OPP. Genes belonging to the functional classes of "amino acid biosynthesis and metabolism", "energy metabolism and "putative enzymes" contained a relatively higher number of genes. It was interesting to find the *nap*A, B, D and F genes among the genes in the class of energy metabolism. These genes are some of the components of the *nap *operon that has been identified in many gram negative bacteria [64]. The *E. coli *nap operon (*nap*FDAGHBC) encodes a periplasmic nitrate reductase [65]. The respiratory periplasmic nitrate reductase in denitrifying *P*. sp. Strain G-179 has been reported to support anaerobic growth in the presence of nitrate [66]. Most Nap enzymes consist of a large catalytic sub subunit (NapA) and a small diheme cytochrome *c *(NapB). NapC is a membrane bound tetraheme cytochrome *c *that transfers electrons from the quinol pool in the cytoplasmic membrane to NapAB. NapD is found in the cytoplasm and plays a role in the maturation of the enzyme prior to export [67] and NapF is a non-heme iron-sulfur protein [68]. The downregulation of the nap A, B, D and F genes in this study suggests that after 60 minutes of OPP treatment, *P. aeruginosa *probably maintains aerobic growth. This is in contrast to after 20 minutes of treatment when the nitric oxide reductase gene was upregulated, suggesting a possible transient switch to anaerobic respiration (table 1).

This group also contained several genes involved in valine, leucine and isoleucine degradation. In particular, PA2247 (*bkd*A1), PA2248 (*bkd*A2), and 2250 (*lpd*V) which are involved in the conversion of valine, leucine and isoleucine to alkyl-CoA derivatives that feed into the TCA cycle, pyrimidine metabolism and propanoate metabolism were downregulated. Similarly, the *bkd*A1, *bkd*A2 and *lpd*V genes were downregulated in *P. aeruginosa *after exposure to peracetic acid for 20 minutes [16]. The results of the present study suggest that the synthesis of these amino acids was being inhibited after 60 minutes of OPP treatment with concomitant inhibition of energy production through the TCA cycle. Further, several genes involved in the synthesis of acetylCoA were present in this group. In particular, PA2013 and PA0745 (probable enoylCoA hydratases) involved in the synthesis of acetylCoA from Lysine and butanoate respectively and PA3417 (probable pyruvate dehydrogenase E1 component) which catalyzes the transformation of pyruvate to acetylCoA were downregulated. These results support the fact that energy production through the TCA cycle was being inhibited after 60 minutes of exposure to OPP.

#### Group VI: genes downregulated at 20 and 60 minutes exposure

The most downregulated gene in this group was the ribosome modulation factor gene (*rmf*), which exhibited a fold change of -6.25 after 20 minutes of OPP treatment and -25.9 after 60 minutes. The ribosome modulation factor (RMF) is a ribosome associated protein that is produced by *E. coli *during slow growth at exponential phase [69] and upon entry into stationary phase [70]. RMF is considered a protective factor of ribosomes against stresses such as heat shock, acidic/basic pH and high osmolarity during stationary phase [71]. Mutant stationary phase *E. coli *strains without functional RMF are more susceptible to osmotic stress [72] and heat stress [73]. The production of RMF by stationary phase cells has been linked to the detection of the dimerized form of 70S ribosomes: 100S ribosomes with no translational activity [71,74,75].

In exponentially growing *E. coli *cells not treated with any chemicals and in those treated with acidifying agents, it was found that *rmf *expression was growth rate dependent and there was an inverse relationship between *rmf *expression and growth rate [69,76]. It has been postulated that the function of RMF in slow growing exponential phase cells is to promote more efficient protein synthesis through the inactivation of excess ribosomes, thereby reducing competition for protein synthesis factors [76]. Our results contrast those of previous studies which have reported that *rmf *is expressed in slow growing cells during exponential phase [69,76]. It was surprising that *rmf *expression was downregulated upon both treatment times, but more significantly after 60 minutes, given the fact that OPP-treated *P. aeruginosa *cells in this study were also slow growing during exponential phase. However, more investigation is required to determine the mechanism by which OPP treatment influences transcriptional control, leading to downregulation of the expression of the *rmf *gene in *P. aeruginosa*.

The *rpo*S gene which encodes RpoS, an alternative sigma factor of RNA ploymerase was also downregulated after both exposure times. RpoS is known to participate in the stress response of both *E. coli *and *P. aeruginosa *[77,78]. Although RpoS is more widely considered a global regulator in a complex regulatory network that controls the expression of several stationary-phase inducible genes, it has been demonsrated that RpoS also acts as a master regulator of gene expression in exponentially growing *E. coli *cells exposed to osmotic stress [79]. Further, a previous study revealed that cell viability was slightly decreased in *E. coli *cells containing mutations in *rmf *and *rpo*S [80]. The downregulation of the *rmf *and *rpo*S genes may therefore be indicative of the mechanism of action by which OPP causes a growth inhibition in *P. aeruginosa*.

This group also contained four genes that are involved in the *P. aeruginosa *heat shock response: *grp*E, *hsl*U, *hsl*V and *htp*G. In contrast to these results, the HtpG and GrpE proteins have been reported to be upregulated in *P. putida *exposed to phenol and to hydrochloric acid [32,81]. The *hsl*VU operon encodes two heat shock proteins, HslV and HslU that function together as an ATP-dependent protease [82]. The *hsl*UV operon was upregulated in *E. coli *after exposure to acid stress for 10 minutes [81]. This result suggests that transcriptional regulation of the expression of these heat shock proteins during stress may vary depending on the nature of the environmental stress.

### Validation of microarray data using real-time PCR

In order to validate the relative transcript levels obtained by the microarray analysis, we employed quantitative real-time PCR on six genes. These genes were selected because they displayed a wide range of mRNA level changes (-25- to 6-fold). Table 2 indicates that our microarray results were in agreement with quantitative real-time PCR analyses of the selected genes.

**Table 2:** Transcript level comparison of *P. aeruginosa *genes between real-time PCR and microarray analyses

Gene	^a^mRNA level change with microarray		^b^mRNA level change with real-time PCR		Forward primer sequence (5'-3')	Reverse primer sequence (5'-3')
			
	Fold change		Fold change			
			
	20 min	60 min	20 min	60 min		
PA4243	2.914	6.799	3.605 (± 1.55)	7.378 (± 0.7)	ATGGCTAAGCAAGGTGCTCTCTCT	ACGATGATCGCCAGGAACAGGAAA
PA2193	-2.268		-1.967 (± 1.06)		TGAACGTCAACACGATATCCAGCC	ATTGAGCACGTTGAGCACGGTCT
PA1173		-3.077		-3.03 (± 5.64)	ATCGACAAGGACAGCAACAAGTGC	GTCCATGTAGTGGGTGATGCTGAT
PA3049	-6.25	-25.907	-10.196 (± 0.14)	-48.503 (± 0.35)	TCGTGATCTTTGTCCGTTCACCCA	CGTGCTGGAGTTGATTGAGACGTT
PA3724	-2.762	-10.256	-5.856 (± 0.21)	-14.928 (± 1.27)	TCATCACCGTCGACATGAACAGCA	AGTCCCGGTACAGTTTGAACACCA
PA3622	-2.653	-2.967	-1.954 (± 1.95)	-5.924 (± 1.60)	TGACCACGATGATGAAGTGCTCCT	TTGGAAGAGAAGGAAGTGGTGGCT
^c^PA3001	1.00	1.00	1.00	1.00	GCACCATCACCATCGACGAAGAAA	TCTTGATGCCGTACTGGGTGTAGT

The real time PCR results are the mean of three biological replicates with three technical replicates for each gene. The microarray results are the mean of four replicates of each gene.

^a^The microarray results are the mean of four replicates of each gene.

^b^The real time PCR results are the mean of three biological replicates with three technical replicates for each gene.

^c^Internal control: glyceraldehyde-3-phosphate dehydrogenase

## Conclusion

The present study represents the first genome-wide response of *P. aeruginosa *exposed to 0.82 mM OPP. The results from this study indicate that after 20 minutes of OPP exposure, genes involved in anaerobic metabolism and swarming motility were upregulated. We suggest that *P. aeruginosa *undergoes a switch to denitrification as indicated by downregulation of cyanide production which is indicative of anaerobic respiration. OPP treatment also caused the downregulation of the genes encoding the ribosome modulation factor (*rmf*) and an alternative sigma factor (*rpo*S) of RNA polymerase which have been linked to decreases in cell viability when mutated. The repression of these genes may be contributory to the growth inhibition observed after *P. aeruginosa *was exposed to OPP and may reflect the mechanism of action by which OPP reduces the viability of *P. aeruginosa *cells, leading to the observed growth inhibition. We suspect that the continuous marked upregulation of translation after both 20 and 60 minutes and of amino acid biosynthesis following 60 minutes exposure to OPP are consequential responses to combat this growth repression. Our results suggest that these responses may involve the upregulation of genes involved in the synthesis of membrane transport and virulence proteins and also proteins involved in the maintainanceof the integrity of the cell membrane. In addition, after 60 minutes of OPP treatment, the adaptive response to OPP treatment may involve cell wall modification evidenced by the upregulation of lipopolysaccharide biosynthesis genes. It is worth noting that in contrast to the results of this study, we have observed that in *S. aureus*, OPP treatment led to downregulation of amino acid anabolism in general and specifically lysine and diaminopimelic acid (DAP) biosynthesis genes (unpublished data). It is therefore apparent that OPP exerts differential effects on amino acid metabolism in *P. aeruginosa *and *S. aureus*.

This gene expression profile can now be employed to more profoundly understand the mechanisms by which OPP exerts a killing effect on *P. aeruginosa *and how this organism develops resistance to phenolic disinfectants in general and to OPP in particular. The information from this study provides useful information that will benefit further research on the toxicogenomic impact of phenolic biocides on *P. aeruginosa*. Further, considering that multicellular behavior in bacteria such as swarming motility is an adaptation to environmental stress, it will be interesting to investigate the comparative response of sessile cells versus cells exhibiting swarming motility to OPP treatment.

## Methods

### Bacterial growth and treatment with OPP

*Pseudomonas aeruginosa *PAO1 was grown at 37°C for 17 hours on Luria-Bertani (LB) agar. An isolated colony was inoculated into 100 ml of sterilized LB broth (10 g of tryptone, 5 g of yeast extract and 10 g of sodium chloride per liter) and incubated overnight for 17 hours at 37°C with shaking at 250 rpm. A 1:100 dilution of the culture was performed using pre-warmed LB broth. The diluted culture was incubated at 37°C with shaking at 250 rpm until a final optical density (OD_600_) of 0.8 (early logarithmic phase) was attained. A further 1:10 dilution was performed using LB broth and incubated at 37°C with shaking at 250 rpm. When the OD_600 _of the 1:100 dilution reached 0.8, the culture was incubated at 37°C with shaking at 250 rpm with various concentrations of OPP (Sigma-Aldrich, Inc., St Louis, MO) and the OD_600 _of the growth culture was determined at intervals of ten minutes for a total time of 60 minutes. A concentration of 0.82 mM OPP, with treatment times of 20 and 60 minutes were targeted for this study.

### RNA isolation

Total RNA was extracted after 20 and 60 minutes incubation with 0.82 mM OPP and without OPP (control). The RNA extraction procedure was carried out using the RNeasy mini kit (Qiagen, Inc., Valencia, CA) according to the manufacturer's instructions. Briefly, 1 ml of bacterial culture was added to 2 ml of RNAprotect bacteria reagent (Qiagen, Inc., Valencia, CA). Centrifugation (5000 g for 10 minutes) of the mixture was performed to precipitate the cells. The harvested cells were incubated in TE buffer with 1 mg/ml lysozyme (Fisher Scientific, Pittsburgh, PA). Total RNA was eluted in 50 ml of RNase free water (Ambion Inc., Austin Texas) using kit supplied columns containing silica gel membranes. The quantity of eluted RNA was determined using the NanoDrop spectrophotometer (NanoDrop Technologies, Inc., Wilmington, DE). RNA quality was examined using the RNA 6000 Nano Labchip with an Agilent 2100 Bioanalyzer (Agilent Technologies, Palo Alto, CA).

### cDNA synthesis and labeling

cDNA was synthesized from 12 μg of total RNA using random primers (Invitrogen, Carlsbad, CA)and SuperScript II reverse transcriptase (Invitrogen, Carlsbad, CA) following the Affymetrix *P. aeruginosa *GeneChip arrays protocol (Affymetrix, Inc., Santa Clara, CA). Spike controls containing RNA transcripts from several *Bacillus subtilis *genes were included in the RNA mixtures as internal controls to monitor the efficiency of labeling, hybridization and staining. The reaction mixture was incubated at 25° for 10 minutes, 37°C for 60 minutes and 42°C for 60 minutes followed by inactivation of the enzyme at 70°C for 10 minutes. Purification of cDNA was carried out using the QIAquick PCR purification kit (Qiagen, Inc., Valencia, CA). The cDNA was fragmented at 37°C for 10 minutes in a reaction mixture consisting of purified cDNA and DNase I (Roche Applied Science, Indianapolis) in One-Phor-All buffer (Invitrogen, Carlsbad, CA) in the order of 0.06 U DNase/μl of cDNA. The quality of fragmented cDNA was examined using the RNA 6000 Nano Labchip with an Agilent 2100 Bioanalyzer (Agilent Technologies, Palo Alto, CA). Labeling of the 3' termini of fragmented cDNA was performed using the Enzo BioArray terminal labeling kit with Biotin-ddUTP (Enzo Life Sciences, Inc., Farmingdale, NY).

### Hybridization, processing and scanning

Array hybridization and processing were carried out according to instructions provided in the affymetrix expression analysis technical manual: chapters 5 and 6 [83]. The hybridization solution consisted of the fragmented/labeled cDNA, B2 control oligonucleotide, MES hybridization buffer, bovine serum albumin and dimethyl sulfoxide (DMSO) in a final volume of 200 μl. The mixture was hybridized onto *P. aeruginosa *GeneChip arrays (Affymetrix, Inc., Santa Clara, CA) at 50°C for 16 hours with tumbling. The arrays were washed and stained using the Affymetrix GeneChip hybridization, wash and stain kit containing the stain cocktails 1 and 2 and the array holding buffer. The array staining and washing process was performed using the GeneChip Fluidics station 450 (Affymetrix). Processed arrays were scanned with the Affymetrix GeneChip Scanner 3000.

### Data analysis

Data analysis was carried out using the Affymetrix GeneChip Operating Software (GCOS), version 1.0 and GeneSpring Version 7.3 (Agilent Technologies). The following parameters were employed for expression analysis using GCOS: α_1 _= 0.04, α_2 _= 0.06, τ = 0.015 and target signal was scaled to 150. Genes that were assigned "absent calls" from 50% or more of the replicates in GeneSpring were not included in the analysis. Gene expression changes with statistical significance were identified by 1-way ANOVA (*p *cutoff value = 0.05). Fold changes were calculated as the ratios between the signal averages of four untreated (control) and four OPP-treated (experimental) cultures. Genes with a two-fold or more induction or repression were used in this analysis.

### Real-Time PCR analysis

Transcript level changes obtained from microarray analysis (six genes) were evaluated using quantitative real-time PCR. The genes and primer sequences employed for the real-time PCR analysis are listed in table 2. The housekeeping gene, glyceraldehyde-3-phosphate dehydrogenase (PA3001) was used as an endogenous control. Real-time PCR was performed using the iCycler iQ Real-Time PCR Detection System with iScript cDNA Synthesis Kit and IQ SYBR Green Supermix (BioRad Laboratories, Inc., Hercules, CA). For each gene, three biological replicates and three technical replicates were employed. Reaction mixtures were incubated for 3 minutes at 95.0°C, followed by 40 cycles of 10 seconds at 95.0°C, 30 seconds at 55.0°C, and 20 seconds at 72.0°C. PCR efficiencies were also derived from standard curve slopes in the iCycler software v. 3.1 (BioRad Laboratories, Inc., Hercules, CA). To evaluate PCR specificity, melt curve analysis was performed and this resulted in single primer-specific melting temperatures. In this report, relative quantification based on the relative expression of a target gene versus the glyceraldehyde-3-phosphate dehydrogenase gene was utilized to determine the transcript level changes.

## Authors' contributions

CWN performed microarray experiments, and data analysis, and drafted the manuscript. HJ performed microarray experiments, and data analysis, and reviewed the manuscript. FT initiated and supervised the study and reviewed the manuscript. WEB reviewed the manuscript.

## Supplementary Material

Additional File 1 **The raw data representing the entire genome (5900 genes) under control conditions (0 minutes) and experimental (after 20 and 60 minutes exposure to 0.82 mM OPP)**. This data has also been deposited in NCBI's Gene Expression Omnibus  and can be accessed through the GEO series accession number: GSE10604 Click here for file

Additional File 2 **List of 509 functionally classified *P. aeruginosa *genes that were significantly up and downregulated after 20 and 60 minutes of OPP exposure**. The genes were categorized into six groups based on their transcription patterns. Click here for file
