# Supplementary material for: Toxicogenomic response of Pseudomonas aeruginosa to ortho-phenylphenol
Source: BMC Genomics. 2008 Oct 10;9:473. doi: 10.1186/1471-2164-9-473 (PMC2577666; doi:10.1186/1471-2164-9-473)
Supplement: Additional File 2 — List of 509 functionally classified P. aeruginosa genes that were significantly up and downregulated after 20 and 60 minutes of OPP exposure. The genes were categorized into six groups based on their transcription patterns. [file 1471-2164-9-473-S2.doc]

**Additional file 2. List of 509 functionally classified *P. aeruginosa* genes that were**

**significantly up and downregulated after 20 and 60 minutes of OPP exposure.**

| **Affymetrix ORF #** | **Probe ID** | **20 minutes** | | **60 minutes** | | **Description** | **Symbol** | **Functional class** |
| --- | --- | --- | --- | --- | --- | --- | --- | --- |
|  |  | **Fold change** | **P value** | **Fold change** | **P value** |  |  |  |
| **Group I: Upregulation (20min) - Upregulation (60 min) 150genes** | | | | | | | | |
| PA1964_at | PA1964 | 2.029 | 0.0112 | 2.381 | 0.0112 | probable ATP-binding component of ABC transporter |  | Transport of small molecules |
| PA4616_at | PA4616 | 2.098 | 0.00236 | 2.357 | 0.00236 | probable c4-dicarboxylate-binding protein |  | Transport of small molecules |
| PA2760_at | PA2760 | 2.152 | 0.0416 | 2.94 | 0.0416 | probable outer membrane protein precursor |  | Transport of small molecules |
| PA4687_hitA_at | PA4687 | 2.219 | 0.00808 | 2.069 | 0.00808 | ferric iron-binding periplasmic protein HitA | *hit*A | Transport of small molecules |
| PA4247_rplR_at | PA4247 | 2.007 | 0.0226 | 2.648 | 0.0226 | 50S ribosomal protein L18 | *rpl*R | Translation, post-translational modification, degradation |
| PA4254_rpsQ_at | PA4254 | 2.044 | 0.0382 | 4.803 | 0.0382 | 30S ribosomal protein S17 | *rps*Q | Translation, post-translational modification, degradation |
| PA2743_infC_at | PA2743 | 2.044 | 0.000327 | 2.634 | 0.000327 | translation initiation factor IF-3 | *inf*C | Translation, post-translational modification, degradation |
| PA4274_rplK_at | PA4274 | 2.052 | 0.00398 | 4.284 | 0.00398 | 50S ribosomal protein L11 | *rpl*K | Translation, post-translational modification, degradation |
| PA2742_rpmI_at | PA2742 | 2.057 | 0.0118 | 2.647 | 0.0118 | 50S ribosomal protein L35 | *rpm*I | Translation, post-translational modification, degradation |
| PA2970_rpmF_at | PA2970 | 2.061 | 0.00231 | 3.765 | 0.00231 | 50S ribosomal protein L32 | *rpm*F | Translation, post-translational modification, degradation |
| PA4268_rpsL_at | PA4268 | 2.077 | 0.0123 | 4.808 | 0.0123 | 30S ribosomal protein S12 | *rps*L | Translation, post-translational modification, degradation |
| PA4252_rplX_at | PA4252 | 2.127 | 0.00645 | 5.153 | 0.00645 | 50S ribosomal protein L24 | *rpl*X | Translation, post-translational modification, degradation |
| PA2619_infA_at | PA2619 | 2.14 | 0.000526 | 3.644 | 0.000526 | translation initiation factor | *inf*A | Translation, post-translational modification, degradation |
| PA4567_rpmA_at | PA4567 | 2.143 | 0.0269 | 3.511 | 0.0269 | 50S ribosomal protein L27 | *rpm*A | Translation, post-translational modification, degradation |
| PA3802_hisS_at | PA3802 | 2.151 | 0.000281 | 2.848 | 0.000281 | histidyl-tRNA synthetase | *his*S | Translation, post-translational modification, degradation |
| PA4249_rpsH_at | PA4249 | 2.157 | 0.0015 | 4.201 | 0.0015 | 30S ribosomal protein S8 | *rps*H | Translation, post-translational modification, degradation |
| PA4264_rpsJ_at | PA4264 | 2.174 | 7.74E-05 | 4.346 | 7.74E-05 | 30S ribosomal protein S10 | *rps*J | Translation, post-translational modification, degradation |
| PA4266_fusA1_at | PA4266 | 2.18 | 0.012 | 3.88 | 0.012 | elongation factor G | *fus*A1 | Translation, post-translational modification, degradation |
| PA4257_rpsC_at | PA4257 | 2.187 | 0.00117 | 5.082 | 0.00117 | 30S ribosomal protein S3 | *rps*C | Translation, post-translational modification, degradation |
| PA2741_rplT_at | PA2741 | 2.193 | 0.0201 | 2.961 | 0.0201 | 50S ribosomal protein L20 | *rpl*T | Translation, post-translational modification, degradation |
| PA3655_tsf_at | PA3655 | 2.197 | 0.017 | 5.495 | 0.017 | elongation factor Ts | *tsf* | Translation, post-translational modification, degradation |
| PA4256_rplP_at | PA4256 | 2.225 | 0.00709 | 4.511 | 0.00709 | 50S ribosomal protein L16 | *rpl*P | Translation, post-translational modification, degradation |
| PA4241_rpsM_at | PA4241 | 2.267 | 0.00896 | 4.8 | 0.00896 | 30S ribosomal protein S13 | *rps*M | Translation, post-translational modification, degradation |
| PA4246_rpsE_at | PA4246 | 2.295 | 0.0219 | 5.002 | 0.0219 | 30S ribosomal protein S5 | *rps*E | Translation, post-translational modification, degradation |
| PA4432_rpsI_at | PA4432 | 2.307 | 0.00037 | 3.69 | 0.00037 | 30S ribosomal protein S9 | *rps*I | Translation, post-translational modification, degradation |
| PA4259_rpsS_at | PA4259 | 2.313 | 0.00353 | 4.99 | 0.00353 | 30S ribosomal protein S19 | *rps*S | Translation, post-translational modification, degradation |
| PA4568_rplU_at | PA4568 | 2.332 | 0.00189 | 4.92 | 0.00189 | 50S ribosomal protein L21 | *rpl*U | Translation, post-translational modification, degradation |
| PA4563_rpsT_at | PA4563 | 2.333 | 8.11E-06 | 4.956 | 8.11E-06 | 30S ribosomal protein S20 | *rps*T | Translation, post-translational modification, degradation |
| PA4242_rpmJ_at | PA4242 | 2.36 | 0.000606 | 4.381 | 0.000606 | 50S ribosomal protein L36 | *rpm*J | Translation, post-translational modification, degradation |
| PA4239_rpsD_at | PA4239 | 2.4 | 0.00365 | 4.49 | 0.00365 | 30S ribosomal protein S4 | *rps*D | Translation, post-translational modification, degradation |
| PA4433_rplM_at | PA4433 | 2.401 | 0.0021 | 5.088 | 0.0021 | 50S ribosomal protein L13 | *rpl*M | Translation, post-translational modification, degradation |
| PA4260_rplB_at | PA4260 | 2.416 | 0.00508 | 4.16 | 0.00508 | 50S ribosomal protein L2 | *rpl*B | Translation, post-translational modification, degradation |
| PA4665_prfA_at | PA4665 | 2.426 | 0.00398 | 2.774 | 0.00398 | peptide chain release factor 1 | *prf*A | Translation, post-translational modification, degradation |
| PA4932_rplI_at | PA4932 | 2.442 | 0.0124 | 5.756 | 0.0124 | 50S ribosomal protein L9 | *rpl*I | Translation, post-translational modification, degradation |
| PA4261_rplW_at | PA4261 | 2.462 | 0.000224 | 4.879 | 0.000224 | 50S ribosomal protein L23 | *rpl*W | Translation, post-translational modification, degradation |
| PA4245_rpmD_at | PA4245 | 2.482 | 0.00662 | 5.73 | 0.00662 | 50S ribosomal protein L30 | *rpm*D | Translation, post-translational modification, degradation |
| PA3656_rpsB_at | PA3656 | 2.543 | 0.00953 | 7.267 | 0.00953 | 30S ribosomal protein S2 | *rps*B | Translation, post-translational modification, degradation |
| PA3162_rpsA_at | PA3162 | 2.608 | 0.00303 | 4.75 | 0.00303 | 30S ribosomal protein S1 | *rps*A | Translation, post-translational modification, degradation |
| PA4935_rpsF_at | PA4935 | 2.616 | 0.00138 | 5.632 | 0.00138 | 30S ribosomal protein S6 | *rps*F | Translation, post-translational modification, degradation |
| PA4672_at | PA4672 | 2.662 | 0.00229 | 4.295 | 0.00229 | peptidyl-tRNA hydrolase |  | Translation, post-translational modification, degradation |
| PA4273_rplA_at | PA4273 | 2.725 | 0.0349 | 6.018 | 0.0349 | 50S ribosomal protein L1 | *rpl*A | Translation, post-translational modification, degradation |
| PA4263_rplC_at | PA3818 | 2.746 | 0.000725 | 2.564 | 0.000725 | 50S ribosomal protein L3 | *rpl*C | Translation, post-translational modification, degradation |
| PA4744_infB_at | PA4744 | 2.783 | 0.0134 | 3.826 | 0.0134 | translation initiation factor IF-2 | *inf*B | Translation, post-translational modification, degradation |
| PA4258_rplV_at | PA4258 | 2.791 | 0.0219 | 5.199 | 0.0219 | 50S ribosomal protein L22 | *rpl*V | Translation, post-translational modification, degradation |
| PA4741_rpsO_at | PA4741 | 2.791 | 0.00106 | 5.559 | 0.00106 | 30S ribosomal protein S15 | *rps*O | Translation, post-translational modification, degradation |
| PA4934_rpsR_at | PA4934 | 2.894 | 0.000276 | 6.619 | 0.000276 | 30S ribosomal protein S18 | *rps*R | Translation, post-translational modification, degradation |
| PA4255_rpmC_at | PA4255 | 2.927 | 0.00331 | 6.655 | 0.00331 | 50S ribosomal protein L29 | *rpm*C | Translation, post-translational modification, degradation |
| PA5569_rnpA_at | PA5569 | 2.983 | 0.00301 | 5.629 | 0.00301 | ribonuclease P protein component | *rnp*A | Translation, post-translational modification, degradation |
| PA3745_rpsP_at | PA3745 | 3.289 | 0.00403 | 5.416 | 0.00403 | 30S ribosomal protein S16 | *rps*P | Translation, post-translational modification, degradation |
| PA0527_dnr_at | PA0527 | 2.449 | 0.00214 | 6.224 | 0.00214 | transcriptional regulator Dnr | *dnr* | Transcriptional regulators |
| PA5274_rnk_at | PA5274 | 2.704 | 0.00501 | 3.799 | 0.00501 | nucleoside diphosphate kinase regulator | *rnk* | Transcriptional regulators |
| PA0839_at | PA5046 | 2.84 | 0.00871 | 4.349 | 0.00871 | probable transcriptional regulator |  | Transcriptional regulators |
| PA4742_truB_at | PA4742 | 2.199 | 0.000508 | 2.932 | 0.000508 | tRNA pseudouridine 55 synthase | *tru*B | Transcription, RNA processing and degradation |
| PA4740_pnp_at | PA4740 | 2.286 | 0.00345 | 3.392 | 0.00345 | polyribonucleotide nucleotidyltransferase | *pnp* | Transcription, RNA processing and degradation |
| PA0770_rnc_at | PA0770 | 2.549 | 0.0107 | 2.399 | 0.0107 | ribonuclease III | *rnc* | Transcription, RNA processing and degradation |
| PA5239_rho_at | PA5239 | 2.609 | 0.00747 | 3.95 | 0.00747 | transcription termination factor Rho | *rho* | Transcription, RNA processing and degradation |
| PA4275_nusG_at | PA4275 | 2.723 | 2.60E-06 | 4.747 | 2.60E-06 | transcription antitermination protein NusG | *nus*G | Transcription, RNA processing and degradation |
| PA4262_rplD_at | PA4262 | 2.742 | 0.00162 | 5.999 | 0.00162 | 50S ribosomal protein L4 | *rpl*D | Transcription, RNA processing and degradation |
| PA3743_trmD_at | PA3743 | 2.838 | 0.000602 | 0.0975 | 0.000602 | tRNA (guanine-N1)-methyltransferase | *trm*D | Transcription, RNA processing and degradation |
| PA3744_rimM_at | PA3744 | 3.301 | 9.30E-05 | 6.039 | 9.30E-05 | 16S rRNA processing protein | *rim*M | Transcription, RNA processing and degradation |
| PA3768_at | PA3768 | 2.522 | 0.02 | 3.267 | 0.02 | probable metallo-oxidoreductase |  | Putative enzymes |
| PA2939_at | PA2939 | 3.201 | 0.000494 | 0.294 | 0.000494 | probable aminopeptidase |  | Putative enzymes |
| PA4276_secE_at | PA4276 | 2.274 | 2.07E-05 | 4.198 | 2.07E-05 | secretion protein SecE | *sec*E | Protein secretion/export apparatus |
| PA4854_purH_at | PA4854 | 2.081 | 0.0119 | 3.62 | 0.0119 | phosphoribosylaminoimidazolecarboxamide formyltransferase | *pur*H | Nucleotide biosynthesis and metabolism |
| PA1796_folD_at | PA1796 | 2.106 | 0.00115 | 2.502 | 0.00115 | 5,10-methylene-tetrahydrofolate dehydrogenase / cyclohydrolase | *fol*D | Nucleotide biosynthesis and metabolism |
| PA5336_gmk_at | PA5336 | 2.915 | 0.00567 | 3.054 | 0.00567 | guanylate kinase | *gmk* | Nucleotide biosynthesis and metabolism |
| PA4528_pilD_at | PA4528 | 2.144 | 0.014 | 2.65 | 0.014 | type 4 prepilin peptidase PilD | *pil*D | Motility & Attachment |
| PA5041_pilP_at | PA5041 | 2.169 | 0.00817 | 2.232 | 0.00817 | type 4 fimbrial biogenesis protein PilP | *pil*P | Motility & Attachment |
| PA0410_pilI_at | PA0024 | 2.188 | 0.0484 | 2.267 | 0.0484 | twitching motility protein PilI | *pil*I | Motility & Attachment |
| PA5042_pilO_at | PA5042 | 2.26 | 0.000345 | 2.056 | 0.000345 | type 4 fimbrial biogenesis protein PilO | *pil*O | Motility & Attachment |
| PA4527_pilC_at | PA4527 | 2.27 | 0.00205 | 2.678 | 0.00205 | still frameshift type 4 fimbrial biogenesis protein PilC | *pil*C | Motility & Attachment |
| PA5043_pilN_at | PA5043 | 2.34 | 0.000628 | 2.914 | 0.000628 | type 4 fimbrial biogenesis protein PilN | *pil*N | Motility & Attachment |
| PA5044_pilM_at | PA5044 | 2.893 | 0.00702 | 3.12 | 0.00702 | type 4 fimbrial biogenesis protein PilM | *pil*M | Motility & Attachment |
| PA4688_hitB_at | PA4688 | 2.282 | 0.0261 | 2.513 | 0.0261 | iron (III)-transport system permease HitB | *hit*B | Membrane proteins |
| PA4747_secG_at | PA4747 | 2.294 | 0.00628 | 3.795 | 0.00628 | secretion protein SecG | *sec*G | Membrane proteins |
| PA4243_secY_at | PA4243 | 2.914 | 9.26E-05 | 6.799 | 9.26E-05 | secretion protein SecY | *sec*Y | Membrane proteins |
| PA4940_at | PA4940 | 2.035 | 0.000163 | 2.633 | 0.000163 | conserved hypothetical protein |  | Membrane proteins |
| PA5244_at | PA5244 | 2.227 | 0.00308 | 2.299 | 0.00308 | conserved hypothetical protein |  | Membrane proteins |
| PA4933_at | PA4933 | 2.551 | 0.0196 | 6.245 | 0.0196 | hypothetical protein |  | Membrane proteins |
| PA3278_at | PA3278 | 2.782 | 0.0041 | 3.127 | 0.0041 | hypothetical protein |  | Membrane proteins |
| PA3966_at | PA3966 | 3.465 | 0.00221 | 3.777 | 0.00221 | hypothetical protein |  | Membrane proteins |
| PA5130_at | PA5130 | 2.005 | 0.0218 | 3.737 | 0.0218 | conserved hypothetical protein |  | Hypothetical, unclassified, unknown |
| PA4852_at | PA4852 | 2.007 | 0.00443 | 4.168 | 0.00443 | conserved hypothetical protein |  | Hypothetical, unclassified, unknown |
| ig_1047549_1046911_at | 1036911 | 2.032 | 0.00535 | 3.019 | 0.00535 | Intergenic region between PA0981 and PA0982, 1046911-1047549, (-) strand |  | Hypothetical, unclassified, unknown |
| PA4005_at | PA4005 | 2.043 | 0.0201 | 2.569 | 0.0201 | conserved hypothetical protein |  | Hypothetical, unclassified, unknown |
| PA5335_at | PA5335 | 2.077 | 0.00311 | 2.7 | 0.00311 | 30S ribosomal protein S12 |  | Hypothetical, unclassified, unknown |
| PA0663_at | PA0663 | 2.108 | 0.00131 | 3.142 | 0.00131 | hypothetical protein |  | Hypothetical, unclassified, unknown |
| PA0734_i_at | PA0734 | 2.11 | 0.00402 | 2.734 | 0.00402 | hypothetical protein |  | Hypothetical, unclassified, unknown |
| PA0563_at | PA0563 | 2.131 | 0.00398 | 2.904 | 0.00398 | conserved hypothetical protein |  | Hypothetical, unclassified, unknown |
| Pae_tRNA_Gln_s_at | Genome | 2.139 | 0.000487 | 3.929 | 0.000487 | tRNA_Glutamine, 5238277-5238351 (+) strand |  | Hypothetical, unclassified, unknown |
| PA3981_at | PA3981 | 2.153 | 0.0433 | 3.216 | 0.0433 | conserved hypothetical protein |  | Hypothetical, unclassified, unknown |
| PA4968_at | PA4968 | 2.181 | 0.0199 | 2.945 | 0.0199 | conserved hypothetical protein |  | Hypothetical, unclassified, unknown |
| PA5492_at | PA5492 | 2.203 | 0.0466 | 2.577 | 0.0466 | conserved hypothetical protein |  | Hypothetical, unclassified, unknown |
| PA4006_at | PA4006 | 2.204 | 0.00144 | 2.963 | 0.00144 | hypothetical protein |  | Hypothetical, unclassified, unknown |
| PA2630_at | PA2630 | 2.217 | 0.0178 | 3.835 | 0.0178 | conserved hypothetical protein |  | Hypothetical, unclassified, unknown |
| PA4459_at | PA4459 | 2.226 | 0.0032 | 2.773 | 0.0032 | conserved hypothetical protein |  | Hypothetical, unclassified, unknown |
| Pae_tRNA_Ala_f_at | Genome | 2.249 | 0.00123 | 3.152 | 0.00123 | tRNA_Alanine, 723801-723876 (+) strand |  | Hypothetical, unclassified, unknown |
| PA2453_at | PA2453 | 2.253 | 0.000158 | 2.909 | 0.000158 | hypothetical protein |  | Hypothetical, unclassified, unknown |
| Pae_tRNA_Ile_f_at | Genome | 2.258 | 0.00137 | 3.179 | 0.00137 | tRNA_Isoleucine, 723696-723772 (+) strand |  | Hypothetical, unclassified, unknown |
| PA2971_at | PA2971 | 2.275 | 0.00189 | 3.264 | 0.00189 | conserved hypothetical protein |  | Hypothetical, unclassified, unknown |
| PA4888_at | PA4888 | 2.286 | 0.0202 | 2.059 | 0.0202 | conserved hypothetical protein |  | Hypothetical, unclassified, unknown |
| ig_188448_189120_at | 178448 | 2.33 | 0.0142 | 2.633 | 0.0142 | Intergenic region between PA0194 and PA0195, 188448-189120, (+) strand |  | Hypothetical, unclassified, unknown |
| Pae_tRNA_Phe_f_at | Genome | 2.382 | 0.00156 | 2.735 | 0.00156 | tRNA_Phenylalanine, 5798560-5798635 (+) strand |  | Hypothetical, unclassified, unknown |
| PA0579_rpsU_at | PA0579 | 2.402 | 7.98E-05 | 4.407 | 7.98E-05 | 30S ribosomal protein S21 | *rps*U | Hypothetical, unclassified, unknown |
| PA3982_at | PA3982 | 2.42 | 0.0105 | 3.091 | 0.0105 | conserved hypothetical protein |  | Hypothetical, unclassified, unknown |
| ig_991198_991830_at | 981198 | 2.425 | 0.0292 | 3.805 | 0.0292 | Intergenic region between PA0961 and PA0962, 991198-991830, (+) strand |  | Hypothetical, unclassified, unknown |
| Pae_tRNA_Trp_f_at | Genome | 2.528 | 0.00156 | 4.288 | 0.00156 | tRNA_Tryptophan, 4784184-4784259 (-) strand |  | Hypothetical, unclassified, unknown |
| PA4004_at | PA4004 | 2.543 | 0.00394 | 2.991 | 0.00394 | conserved hypothetical protein |  | Hypothetical, unclassified, unknown |
| PA0769_at | PA0769 | 2.579 | 0.000237 | 2.367 | 0.000237 | hypothetical protein |  | Hypothetical, unclassified, unknown |
| Pae_tRNA_Val_f_at | Genome | 2.726 | 0.00238 | 3.508 | 0.00238 | tRNA_Valine , 3650815-3650890 (-) strand |  | Hypothetical, unclassified, unknown |
| PA0045_at | PA0045 | 2.804 | 0.00424 | 4.723 | 0.00424 | hypothetical protein |  | Hypothetical, unclassified, unknown |
| PA4746_at | PA4746 | 2.838 | 0.00145 | 3.807 | 0.00145 | conserved hypothetical protein |  | Hypothetical, unclassified, unknown |
| Pae_tRNA_Gly_s_at | Genome | 2.931 | 0.00294 | 7.245 | 0.00294 | tRNA_Glycine, 4785688-4785761 (-) strand |  | Hypothetical, unclassified, unknown |
| Pae_tRNA_Leu_s_at | Genome | 2.98 | 0.00419 | 4.302 | 0.00419 | tRNA_Leucine, 5541830-5541916 (+) strand |  | Hypothetical, unclassified, unknown |
| PA3967_at | PA3967 | 3.059 | 0.000769 | 3.263 | 0.000769 | hypothetical protein |  | Hypothetical, unclassified, unknown |
| PA4753_at | PA4753 | 3.06 | 0.000241 | 3.467 | 0.000241 | conserved hypothetical protein |  | Hypothetical, unclassified, unknown |
| PA4359_i_at | PA4359 | 3.249 | 0.0301 | 2.555 | 0.0301 | conserved hypothetical protein |  | Hypothetical, unclassified, unknown |
| PA4918_at | PA4918 | 4.903 | 0.016 | 11.75 | 0.016 | hypothetical protein |  | Hypothetical, unclassified, unknown |
| Pae_tRNA_Asn_s_at | Genome | 8.105 | 0.0398 | 8.486 | 0.0398 | tRNA_Asparagine, 3524012-3524087 (+) strand |  | Hypothetical, unclassified, unknown |
| PA2968_fabD_at | PA2968 | 2.137 | 0.0131 | 3.506 | 0.0131 | malonyl-CoA-[acyl-carrier-protein] transacylase | *fab*D | Fatty acid and phospholipid metabolism |
| PA1609_fabB_at | PA1609 | 2.373 | 0.0147 | 2.855 | 0.0147 | beta-ketoacyl-ACP synthase I | *fab*B | Fatty acid and phospholipid metabolism |
| PA2967_fabG_at | PA2967 | 2.387 | 0.00109 | 3.416 | 0.00109 | 3-oxoacyl-[acyl-carrier-protein] reductase | *fab*G | Fatty acid and phospholipid metabolism |
| PA1610_fabA_at | PA1610 | 2.864 | 0.00295 | 4.071 | 0.00295 | beta-hydroxydecanoyl-ACP dehydrase | *fab*A | Fatty acid and phospholipid metabolism |
| PA5556_atpA_at | PA5556 | 2.202 | 0.00136 | 3.907 | 0.00136 | ATP synthase alpha chain | *atp*A | Energy metabolism |
| PA5491_at | PA5491 | 2.354 | 0.0049 | 2.854 | 0.0049 | probable cytochrome |  | Energy metabolism |
| PA5561_atpI_at | PA5561 | 2.558 | 0.0015 | 2.542 | 0.0015 | ATP synthase protein I | *atp*I | Energy metabolism |
| PA4853_fis_at | PA4853 | 2.019 | 0.0192 | 3.025 | 0.0192 | DNA-binding protein Fis | *fis* | DNA replication, recombination, modification and repair |
| PA0408_pilG_at | PA0408 | 2.294 | 0.0144 | 4.026 | 0.0144 | twitching motility protein PilG | *pil*G | Chemotaxis |
| PA3584_glpD_at | PA3584 | 2.429 | 0.000293 | 7.948 | 0.000293 | glycerol-3-phosphate dehydrogenase | *glp*D | Central intermediary metabolism |
| PA4748_tpiA_at | PA4748 | 2.541 | 0.000215 | 3.397 | 0.000215 | triosephosphate isomerase | *tpi*A | Central intermediary metabolism |
| PA5570_rpmH_at | PA5570 | 2.658 | 0.0032 | 5.46 | 0.0032 | 50S ribosomal protein L34 | *rpm*H | Central intermediary metabolism |
| PA5046_at | PA0839 | 2.84 | 0.00915 | 2.742 | 0.00915 | malic enzyme |  | Central intermediary metabolism |
| PA0430_metF_at | PA0430 | 2.902 | 0.0146 | 2.08 | 0.0146 | 5,10-methylenetetrahydrofolate reductase | *met*F | Central intermediary metabolism |
| PA3452_mqoA_at | PA3452 | 2.972 | 0.022 | 3.038 | 0.022 | malate:quinone oxidoreductase | *mqo*A | Central intermediary metabolism |
| PA4003_pbpA_at | PA4003 | 2.032 | 9.09E-06 | 2.33 | 9.09E-06 | penicillin-binding protein 2 | *pbp*A | Cell wall / LPS / capsule |
| PA3646_lpxD_at | PA3646 | 2.548 | 0.00168 | 3.482 | 0.00168 | UDP-3-O-[3-hydroxylauroyl] glucosamine N-acyltransferase | *lpx*D | Cell wall / LPS / capsule |
| PA3645_fabZ_at | PA3645 | 2.854 | 2.25E-05 | 4.671 | 2.25E-05 | (3R)-hydroxymyristoyl-[acyl carrier protein] dehydratase | *fab*Z | Cell wall / LPS / capsule |
| PA5565_gidA_at | PA5565 | 2.356 | 0.000617 | 2.484 | 0.000617 | glucose-inhibited division protein A | *gid*A | Cell division |
| PA4670_prs_at | PA4670 | 2.08 | 0.00395 | 3.749 | 0.00395 | ribose-phosphate pyrophosphokinase | *prs* | Carbon compound catabolism |
| PA4053_ribE_at | PA4053 | 2.139 | 3.48E-05 | 2.567 | 3.48E-05 | 6,7-dimethyl-8-ribityllumazine synthase | *rib*E | Biosynthesis of cofactors, prosthetic groups and carriers |
| PA0024_hemF_at | PA0410 | 2.188 | 0.0331 | 2.793 | 0.0331 | coproporphyrinogen III oxidase, aerobic | *hem*F | Biosynthesis of cofactors, prosthetic groups and carriers |
| PA2629_purB_at | PA2629 | 2.05 | 0.00478 | 2.636 | 0.00478 | adenylosuccinate lyase | *pur*B | Amino acid biosynthesis and metabolism |
| PA3700_lysS_at | PA3700 | 2.095 | 0.0247 | 3.068 | 0.0247 | lysyl-tRNA synthetase | *lys*S | Amino acid biosynthesis and metabolism |
| PA4846_aroQ1_at | PA4846 | 2.295 | 0.00058 | 3.154 | 0.00058 | 3-dehydroquinate dehydratase | *aro*Q1 | Amino acid biosynthesis and metabolism |
| PA2744_thrS_at | PA2744 | 2.491 | 0.00178 | 3.292 | 0.00178 | threonyl-tRNA synthetase | *thr*S | Amino acid biosynthesis and metabolism |
| PA3537_argF_at | PA3537 | 3.074 | 0.0308 | 3.18 | 0.0308 | ornithine carbamoyltransferase, anabolic | *arg*F | Amino acid biosynthesis and metabolism |
| PA0456_at | PA0456 | 2.117 | 1.15E-05 | 4.102 | 1.15E-05 | probable cold-shock protein |  | Adaptation, protection |
| PA3818_at | PA4263 | 2.746 | 0.00537 | 5.447 | 0.00537 | extragenic suppressor protein SuhB | *Suh*B | Adaptation, protection |
| PA4743_rbfA_at | PA4743 | 2.824 | 0.0177 | 4.063 | 0.0177 | ribosome-binding factor A | *rbf*A | Adaptation, protection |
| PA5117_typA_at | PA5117 | 3.136 | 0.000343 | 5.723 | 0.000343 | regulatory protein TypA | *Typ*A | Adaptation, protection |
| **Group II: Upregulation (20min) - No change (60 min) 13 genes** | | | | | | | | |
| PA3479_rhlA_at | PA3479 | 2.376 | 0.0364 |  |  | rhamnosyltransferase chain A | *rhl*A | Secreted Factors (toxins, enzymes, alginate) |
| PA5446_i_at | PA5446 | 2.871 | 0.00272 |  |  | hypothetical protein |  | Hypothetical, unclassified, unknown |
| PA0526_at | PA0526 | 2.77 | 0.0208 |  |  | hypothetical protein |  | Hypothetical, unclassified, unknown |
| PA0529_at | PA0529 | 2.727 | 0.0295 |  |  | conserved hypothetical protein |  | Hypothetical, unclassified, unknown |
| ig_1255042_1254309_at | 1244309 | 2.125 | 0.0248 |  |  | Intergenic region between PA1221 and PA1222, 1254309-1255042, (-) strand |  | Hypothetical, unclassified, unknown |
| PA0952_at | PA0952 | 2.089 | 0.039 |  |  | hypothetical protein |  | Hypothetical, unclassified, unknown |
| PA0080_at | PA0080 | 2.082 | 0.0089 |  |  | hypothetical protein |  | Hypothetical, unclassified, unknown |
| PA1012_at | PA1012 | 2.012 | 0.00448 |  |  | conserved hypothetical protein |  | Hypothetical, unclassified, unknown |
| PA3334_at | PA3334 | 2.942 | 0.014 |  |  | probable acyl carrier protein |  | Fatty acid and phospholipid metabolism |
| PA0524_norB_at | PA0524 | 3.869 | 0.0456 |  |  | nitric-oxide reductase subunit B | *nor*B | Energy metabolism |
| PA2234_at | PA2234 | 2.137 | 0.0339 |  |  | probable exopolysaccharide transporter |  | Cell wall / LPS / capsule |
| PA0177_at | PA0177 | 2.683 | 0.00294 |  |  | probable purine-binding chemotaxis protein |  | Adaptation, protection |
| PA2761_at | PA2761 | 2.059 | 0.0187 |  |  | hypothetical protein |  | Membrane proteins |
| **Group III: Downregulation (20min) - No change (60 min) 23 genes** | | | | | | | | |
|  |  | Fold change | P value |  |  |  |  |  |
| PA0604_at | PA0604 | -2.203 | 0.0321 |  |  | probable binding protein component of ABC transporter |  | Transport of small molecules |
| PA4542_clpB_at | PA4542 | -2.77 | 0.00253 |  |  | ClpB protein | *clp*B | Translation, post-translational modification, degradation |
| PA3721_at | PA3721 | -3.125 | 0.00741 |  |  | probable transcriptional regulator |  | Transcriptional regulators |
| PA5312_at | PA5312 | -2.519 | 0.0209 |  |  | probable aldehyde dehydrogenase |  | Putative enzymes |
| PA0779_at | PA0779 | -3.773 | 0.000778 |  |  | probable ATP-dependent protease |  | Putative enzymes |
| ig_5207621_5208463_at | 5197621 | -2.061 | 0.0112 |  |  | Intergenic region between PA4674 and PA4675, 5207621-5208463, (+) strand |  | Hypothetical, unclassified, unknown |
| PA3270_at | PA3270 | -2.075 | 0.00914 |  |  | hypothetical protein |  | Hypothetical, unclassified, unknown |
| PA3952_at | PA3952 | -2.119 | 0.00132 |  |  | hypothetical protein |  | Hypothetical, unclassified, unknown |
| PA1203_at | PA1203 | -2.336 | 0.0424 |  |  | hypothetical protein |  | Hypothetical, unclassified, unknown |
| PA4661_at | PA4661 | -2.439 | 0.00144 |  |  | hypothetical protein |  | Hypothetical, unclassified, unknown |
| PA4773_at | PA4773 | -4.098 | 0.00343 |  |  | hypothetical protein |  | Hypothetical, unclassified, unknown |
| PA0999_fabH1_at | PA0999 | -2.024 | 0.0274 |  |  | 3-oxoacyl-[acyl-carrier-protein] synthase III | *fab*H1 | Fatty acid and phospholipid metabolism |
| PA4385_groEL_at | PA4385 | -2.16 | 0.00513 |  |  | GroEL protein | *gro*EL | Chaperones & heat shock proteins |
| PA2195_hcnC_at | PA2195 | -2.183 | 0.0437 |  |  | hydrogen cyanide synthase HcnC | *hcn*C | Central intermediary metabolism |
| PA2193_hcnA_at | PA2193 | -2.268 | 0.0324 |  |  | hydrogen cyanide synthase HcnA | *hcn*A | Central intermediary metabolism |
| PA4640_mqoB_at | PA4640 | -2.38 | 0.00881 |  |  | malate:quinone oxidoreductase | *mqo*B | Central intermediary metabolism |
| PA2194_hcnB_at | PA2194 | -2.762 | 0.018 |  |  | hydrogen cyanide synthase HcnB | *hcn*B | Central intermediary metabolism |
| PA4759_dapB_at | PA4759 | -2.075 | 0.00548 |  |  | dihydrodipicolinate reductase | *dap*B | Amino acid biosynthesis and metabolism |
| PA4548_at | PA4548 | -2.5 | 0.000509 |  |  | probable D-amino acid oxidase |  | Amino acid biosynthesis and metabolism |
| PA4356_xenB_at | PA4356 | -2.227 | 0.00385 |  |  | xenobiotic reductase | *xen*B | Adaptation, protection |
| PA0426_mexB_at | PA0426 | -2.146 | 0.0496 |  |  | Resistance-Nodulation-Cell Division (RND) multidrug efflux transporter MexB | *mex*B | Membrane proteins |
| PA3558_at | PA3558 | -2.016 | 0.000294 |  |  | hypothetical protein |  | Membrane proteins |
| PA3369_at | PA3369 | -2.179 | 0.004 |  |  | hypothetical protein |  | Membrane proteins |
| **Group IV: No change(20min) - Upregulation(60 min) 227 genes** | | | | | | | | |
|  |  |  |  | Fold change | P value |  |  |  |
| PA3581_glpF_at | PA3581 |  |  | 4.596 | 0.0458 | glycerol uptake facilitator protein | *glp*F | Transport of small molecules |
| PA1183_dctA_at | PA1183 |  |  | 4.511 | 0.0025 | C4-dicarboxylate transport protein | *dct*A | Transport of small molecules |
| PA3641_at | PA3641 |  |  | 2.974 | 0.0228 | probable amino acid permease |  | Transport of small molecules |
| PA0295_at | PA0295 |  |  | 2.643 | 0.0147 | probable periplasmic polyamine binding protein |  | Transport of small molecules |
| PA4466_at | PA4466 |  |  | 2.286 | 0.00265 | probable phosphoryl carrier protein |  | Transport of small molecules |
| PA0971_tolA_at | PA0971 |  |  | 2.249 | 0.000381 | TolA protein | *tol*A | Transport of small molecules |
| PA4461_at | PA4461 |  |  | 2.24 | 0.00116 | probable ATP-binding component of ABC transporter |  | Transport of small molecules |
| PA5503_at | PA5503 |  |  | 2.229 | 0.000446 | probable ATP-binding component of ABC transporter |  | Transport of small molecules |
| PA0119_at | PA0119 |  |  | 2.151 | 0.0157 | probable dicarboxylate transporter |  | Transport of small molecules |
| PA5217_at | PA5217 |  |  | 2.141 | 0.00709 | probable binding protein component of ABC iron transporter |  | Transport of small molecules |
| PA0280_cysA_at | PA0280 |  |  | 2.018 | 0.0139 | sulfate transport protein CysA | *cys*A | Transport of small molecules |
| PA4272_rplJ_at | PA4272 |  |  | 5.918 | 0.00886 | 50 S ribosomal protein L10 | *rpl*J | Translation, post-translational modification, degradation |
| PA4271_rplL_at | PA4271 |  |  | 5.898 | 0.00319 | 50 S ribosomal protein L17/L12 | *rpl*L | Translation, post-translational modification, degradation |
| PA4253_rplN_at | PA4253 |  |  | 4.714 | 0.0322 | 50 S ribosomal protein L14b/L23e | *rpl*N | Translation, post-translational modification, degradation |
| PA3742_rplS_at | PA3742 |  |  | 3.872 | 0.00842 | 50 s ribosomal protein L19 | *rpl*S | Translation, post-translational modification, degradation |
| PA4251_rplE_at | PA4251 |  |  | 3.659 | 0.0377 | 50S ribosomal protein L5 | *rpl*E | Translation, post-translational modification, degradation |
| PA5316_rpmB_at | PA5316 |  |  | 3.643 | 0.00101 | 50S ribosomal protein L28 | *rpm*B | Translation, post-translational modification, degradation |
| PA4248_rplF_at | PA4248 |  |  | 3.571 | 0.00963 | 50S ribosomal protein L6 | *rpl*F | Translation, post-translational modification, degradation |
| PA2851_efp_at | PA2851 |  |  | 3.516 | 2.08E-05 | translation elongation factor P | *efp* | Translation, post-translational modification, degradation |
| PA2740_pheS_at | PA2740 |  |  | 3.323 | 0.000548 | phenylalanyl-tRNA synthetase, alpha-subunit | *phe*S | Translation, post-translational modification, degradation |
| PA4240_rpsK_at | PA4240 |  |  | 3.283 | 0.0292 | 30 s ribosomal protein S11 | *rps*K | Translation, post-translational modification, degradation |
| PA4265_tufA_s_at | PA4265 |  |  | 3.182 | 0.00461 | elongation factor Tu ; | *tuf*A | Translation, post-translational modification, degradation |
| PA4267_rpsG_at | PA4267 |  |  | 3.104 | 0.0138 | 30 s ribosomal protein S7 | *rps*G | Translation, post-translational modification, degradation |
| PA5049_rpmE_at | PA5049 |  |  | 2.833 | 0.000657 | 50S ribosomal protein L31 | *rpm*E | Translation, post-translational modification, degradation |
| PA0019_def_at | PA0019 |  |  | 2.645 | 0.0441 | polypeptide deformylase |  | Translation, post-translational modification, degradation |
| PA3824_queA_at | PA3824 |  |  | 2.609 | 0.00278 | S-adenosylmethionine:trna ribosyltransferase-isomerase | *que*A | Translation, post-translational modification, degradation |
| PA3262_at | PA3262 |  |  | 2.513 | 0.0384 | probable peptidyl-prolyl cis-trans isomerase |  | Translation, post-translational modification, degradation |
| PA3701_prfB_at | PA3701 |  |  | 2.486 | 0.0344 | peptide chain release factor 2 | *prf*B | Translation, post-translational modification, degradation |
| PA5315_rpmG_at | PA5315 |  |  | 2.39 | 0.0032 | 50S ribosomal protein L33 | *rpm*G | Translation, post-translational modification, degradation |
| PA3657_map_at | PA3657 |  |  | 2.349 | 0.00369 | methionine aminopeptidase |  | Translation, post-translational modification, degradation |
| PA0090_at | PA0090 |  |  | 2.198 | 0.0481 | probable ClpA/B-type chaperone |  | Translation, post-translational modification, degradation |
| PA2739_pheT_at | PA2739 |  |  | 2.142 | 0.0375 | phenylalanyl-tRNA synthetase, beta subunit | *phe*T | Translation, post-translational modification, degradation |
| PA4483_gatA_at | PA4483 |  |  | 2.087 | 0.00998 | Glu-tRNA(Gln) amidotransferase subunit A | *gat*A | Translation, post-translational modification, degradation |
| PA0576_rpoD_at | PA0576 |  |  | 2.513 | 0.0289 | sigma factor RpoD | *rpo*D | Transcriptional regulators |
| PA5437_at | PA5437 |  |  | 2.445 | 0.000807 | probable transcriptional regulator |  | Transcriptional regulators |
| PA0652_vfr_at | PA0652 |  |  | 2.23 | 0.0149 | transcriptional regulator Vfr | *vfr* | Transcriptional regulators |
| PA5337_rpoZ_at | PA5337 |  |  | 2.824 | 0.00806 | RNA polymerase omega subunit | *rpo*Z | Transcription, RNA processing and degradation |
| PA4745_nusA_at | PA4745 |  |  | 2.454 | 0.0264 | N utilization substance protein A | *nus*A | Transcription, RNA processing and degradation |
| PA4052_nusB_at | PA4052 |  |  | 2.362 | 0.0228 | NusB protein | *nus*B | Transcription, RNA processing and degradation |
| PA0903_alaS_at | PA0903 |  |  | 2.099 | 0.0102 | alanyl-tRNA synthetase | *ala*S | Transcription, RNA processing and degradation |
| PA4755_greA_at | PA4755 |  |  | 2.075 | 0.0482 | transcription elongation factor GreA | *gre*A | Transcription, RNA processing and degradation |
| PA4943_at | PA4943 |  |  | 2.663 | 0.0123 | probable GTP-binding protein |  | Putative enzymes |
| PA5005_at | PA5005 |  |  | 2.527 | 0.0182 | probable carbamoyl transferase |  | Putative enzymes |
| PA0386_at | PA0386 |  |  | 2.214 | 0.00112 | probable oxidase |  | Putative enzymes |
| PA3437_at | PA3437 |  |  | 2.148 | 0.00578 | probable short-chain dehydrogenase |  | Putative enzymes |
| PA5008_at | PA5008 |  |  | 2.117 | 0.000927 | hypothetical protein |  | Putative enzymes |
| PA0372_at | PA0372 |  |  | 2.034 | 0.0172 | probable zinc protease |  | Putative enzymes |
| PA0767_lepA_at | PA0767 |  |  | 2.378 | 0.0051 | GTP-binding protein LepA | *lep*A | Protein secretion/export apparatus |
| PA3820_secF_at | PA3820 |  |  | 2.206 | 0.0411 | secretion protein sec F Protein secretion | *sec*F | Protein secretion/export apparatus |
| PA5069_tatB_at | PA5069 |  |  | 2.196 | 0.00448 | translocation protein TatB | *tat*B | Protein secretion/export apparatus |
| PA5298_at | PA5298 |  |  | 3.575 | 0.00118 | xanthine phosphoribosyltransferase |  | Nucleotide biosynthesis and metabolism |
| PA4756_carB_at | PA4756 |  |  | 2.84 | 0.00329 | carbamoylphosphate synthetase large subunit | *car*B | Nucleotide biosynthesis and metabolism |
| PA5321_dut_at | PA5321 |  |  | 2.826 | 0.0035 | deoxyuridine 5'-triphosphate nucleotidohydrolase | *dut* | Nucleotide biosynthesis and metabolism |
| PA3807_ndk_at | PA3807 |  |  | 2.538 | 0.00438 | nucleoside diphosphate kinase | *ndk* | Nucleotide biosynthesis and metabolism |
| PA3654_pyrH_at | PA3654 |  |  | 2.434 | 0.0212 | uridylate kinase | *pyr*H | Nucleotide biosynthesis and metabolism |
| PA3163_cmk_at | PA3163 |  |  | 2.427 | 0.033 | cytidylate kinase | *cmk* | Nucleotide biosynthesis and metabolism |
| PA3763_purL_at | PA3763 |  |  | 2.279 | 0.00792 | phosphoribosylformylglycinamidine synthase | *pur*L | Nucleotide biosynthesis and metabolism |
| PA3527_pyrC_at | PA3527 |  |  | 2.218 | 0.0211 | dihydroorotase | *pyr*C | Nucleotide biosynthesis and metabolism |
| PA5331_pyrE_at | PA5331 |  |  | 2.156 | 0.0166 | orotate phosphoribosyltransferase | *pyr*E | Nucleotide biosynthesis and metabolism |
| PA0590_apaH_at | PA0590 |  |  | 2.152 | 0.0247 | bis(5'-nucleosyl)-tetraphosphatase | *apa*H | Nucleotide biosynthesis and metabolism |
| PA4758_carA_at | PA4758 |  |  | 2.143 | 0.0207 | carbamoyl-phosphate synthase small chain | *car*A | Nucleotide biosynthesis and metabolism |
| PA4314_purU1_at | PA4314 |  |  | 2.086 | 0.00111 | formyltetrahydrofolate deformylase | *pur*U1 | Nucleotide biosynthesis and metabolism |
| PA3480_at | PA3480 |  |  | 2.04 | 0.0185 | probable deoxycytidine triphosphate deaminase |  | Nucleotide biosynthesis and metabolism |
| PA4526_pilB_at | PA4526 |  |  | 2.877 | 0.00493 | type 4 fimbrial biogenesis protein PilB | *pil*B | Motility & Attachment |
| PA1078_flgC_at | PA1078 |  |  | 2.356 | 0.0224 | flagellar basal-body rod protein FlgC | *flg*C | Motility & Attachment |
| PA0291_oprE_at | PA0291 |  |  | 4.797 | 0.00143 | Anaerobically-induced outer membrane porin OprE precursor | *opr*E | Membrane proteins |
| PA3821_secD_at | PA3821 |  |  | 3.516 | 0.00324 | secretion protein SecD | *sec*D | Membrane proteins |
| PA4757_at | PA4757 |  |  | 3.062 | 0.0181 | conserved hypothetical protein |  | Membrane proteins |
| PA4292_at | PA4292 |  |  | 2.955 | 0.0235 | probable phosphate transporter |  | Membrane proteins |
| PA5235_glpT_at | PA5235 |  |  | 2.787 | 0.0238 | glycerol-3-phosphate transporter | *glp*T | Membrane proteins |
| PA5479_gltP_at | PA5479 |  |  | 2.413 | 0.00978 | proton-glutamate symporter | *glt*P | Membrane proteins |
| PA3648_at | PA3648 |  |  | 2.404 | 0.0281 | probable outer membrane protein precursor |  | Membrane proteins |
| PA1305_at | PA1305 |  |  | 2.371 | 0.0128 | hyopthetical |  | Membrane proteins |
| PA5070_tatC_at | PA5070 |  |  | 2.312 | 0.0209 | transport protein TatC | *tat*C | Membrane proteins |
| PA0973_oprL_at | PA0973 |  |  | 2.281 | 0.00672 | Peptidoglycan associated lipoprotein OprL precursor | *opr*L | Membrane proteins |
| PA4455_at | PA4455 |  |  | 2.247 | 0.00226 | probable permease of ABC transporter |  | Membrane proteins |
| PA0162_at | PA0162 |  |  | 2.219 | 0.00407 | probable porin |  | Membrane proteins |
| PA5505_at | PA5505 |  |  | 2.137 | 0.0266 | probable TonB-dependent receptor |  | Membrane proteins |
| PA1051_at | PA1051 |  |  | 2.101 | 0.00316 | probable transporter |  | Membrane proteins |
| PA4451_at | PA4451 |  |  | 3.367 | 0.000317 | conserved hypothetical protein |  | Hypothetical, unclassified, unknown |
| PA5109_at | PA5109 |  |  | 3.352 | 0.0156 | hypothetical protein |  | Hypothetical, unclassified, unknown |
| PA0422_at | PA0422 |  |  | 3.32 | 0.000175 | conserved hypothetical protein |  | Hypothetical, unclassified, unknown |
| PA3822_at | PA3822 |  |  | 3.207 | 2.59E-05 | conserved hypothetical protein |  | Hypothetical, unclassified, unknown |
| PA4998_at | PA4998 |  |  | 3.01 | 0.0127 | conserved hypothetical protein |  | Hypothetical, unclassified, unknown |
| PA4890_at | PA4890 |  |  | 2.935 | 0.00321 | conserved hypothetical protein |  | Hypothetical, unclassified, unknown |
| PA0974_at | PA0974 |  |  | 2.856 | 0.0188 | conserved hypothetical protein |  | Hypothetical, unclassified, unknown |
| PA1768_at | PA1768 |  |  | 2.766 | 0.00612 | hypothetical protein |  | Hypothetical, unclassified, unknown |
| PA0380_i_at | PA0380 |  |  | 2.746 | 0.000141 | conserved hypothetical protein |  | Hypothetical, unclassified, unknown |
| PA4944_at | PA4944 |  |  | 2.645 | 0.000327 | conserved hypothetical protein |  | Hypothetical, unclassified, unknown |
| ig_721556_727608_s_at | 711556 |  |  | 2.632 | 0.00327 | Intergenic region between PA0701 and PA0702, 721556-727608, (+) strand |  | Hypothetical, unclassified, unknown |
| PA2950_at | PA2950 |  |  | 2.607 | 0.00775 | hypothetical protein |  | Hypothetical, unclassified, unknown |
| ig_5541409_5542072_at | 5531409 |  |  | 2.603 | 0.0272 | Intergenic region between PA4957 and PA4958, 5541409-5542072, (+) strand |  | Hypothetical, unclassified, unknown |
| PA4917_at | PA4917 |  |  | 2.6 | 0.032 | hypothetical protein |  | Hypothetical, unclassified, unknown |
| PA0087_at | PA0087 |  |  | 2.525 | 0.0183 | hypothetical protein |  | Hypothetical, unclassified, unknown |
| PA4440_at | PA4440 |  |  | 2.524 | 0.0257 | hypothetical protein |  | Hypothetical, unclassified, unknown |
| PA0856_at | PA0856 |  |  | 2.502 | 0.0201 | hypothetical protein |  | Hypothetical, unclassified, unknown |
| PA0968_at | PA0968 |  |  | 2.492 | 0.00148 | conserved hypothetical protein |  | Hypothetical, unclassified, unknown |
| PA3046_at | PA3046 |  |  | 2.481 | 0.016 | conserved hypothetical protein |  | Hypothetical, unclassified, unknown |
| PA5225_at | PA5225 |  |  | 2.436 | 0.0325 | hypothetical protein |  | Hypothetical, unclassified, unknown |
| PA4441_at | PA4441 |  |  | 2.426 | 0.00335 | hypothetical protein |  | Hypothetical, unclassified, unknown |
| PA0394_at | PA0394 |  |  | 2.406 | 0.0214 | conserved hypothetical protein |  | Hypothetical, unclassified, unknown |
| PA3685_at | PA3685 |  |  | 2.387 | 0.0133 | conserved hypothetical protein |  | Hypothetical, unclassified, unknown |
| PA0201_at | PA0201 |  |  | 2.357 | 0.0048 | hypothetical protein |  | Hypothetical, unclassified, unknown |
| PA0389_at | PA0389 |  |  | 2.322 | 0.00822 | hypothetical protein |  | Hypothetical, unclassified, unknown |
| PA3631_at | PA3631 |  |  | 2.299 | 0.02 | conserved hypothetical protein |  | Hypothetical, unclassified, unknown |
| PA3806_at | PA3806 |  |  | 2.274 | 0.0181 | conserved hypothetical protein |  | Hypothetical, unclassified, unknown |
| PA5463_at | PA5463 |  |  | 2.25 | 6.83E-05 | hypothetical protein |  | Hypothetical, unclassified, unknown |
| PA2753_at | PA2753 |  |  | 2.248 | 0.0111 | hypothetical protein |  | Hypothetical, unclassified, unknown |
| PA4667_at | PA4667 |  |  | 2.199 | 0.0279 | hypothetical protein |  | Hypothetical, unclassified, unknown |
| PA3998_at | PA3998 |  |  | 2.185 | 0.000732 | conserved hypothetical protein |  | Hypothetical, unclassified, unknown |
| PA4684_at | PA4684 |  |  | 2.183 | 0.000934 | hypothetical protein |  | Hypothetical, unclassified, unknown |
| PA3799_at | PA3799 |  |  | 2.171 | 0.0303 | conserved hypothetical protein |  | Hypothetical, unclassified, unknown |
| PA4971_at | PA4971 |  |  | 2.157 | 0.00166 | adenosine diphosphate sugar pyrophosphatase |  | Hypothetical, unclassified, unknown |
| PA4923_at | PA4923 |  |  | 2.148 | 0.00637 | conserved hypothetical protein |  | Hypothetical, unclassified, unknown |
| PA4421_at | PA4421 |  |  | 2.147 | 0.0334 | conserved hypothetical protein |  | Hypothetical, unclassified, unknown |
| PA3009_at | PA3009 |  |  | 2.138 | 0.00501 | hypothetical protein |  | Hypothetical, unclassified, unknown |
| PA4291_at | PA4291 |  |  | 2.138 | 0.043 | hypothetical protein |  | Hypothetical, unclassified, unknown |
| PA1035_at | PA1035 |  |  | 2.115 | 0.0306 | hypothetical protein |  | Hypothetical, unclassified, unknown |
| Pae_tRNA_His_f_at | Genome |  |  | 2.099 | 0.0179 | tRNA_Histidine, 1947729-1947804 (+) strand |  | Hypothetical, unclassified, unknown |
| PA3247_at | PA3247 |  |  | 2.097 | 0.0163 | hypothetical protein |  | Hypothetical, unclassified, unknown |
| PA4029_at | PA4029 |  |  | 2.093 | 0.00753 | conserved hypothetical protein |  | Hypothetical, unclassified, unknown |
| PA1009_at | PA1009 |  |  | 2.084 | 0.0287 | hypothetical protein |  | Hypothetical, unclassified, unknown |
| PA0284_at | PA0284 |  |  | 2.056 | 0.00867 | hypothetical protein |  | Hypothetical, unclassified, unknown |
| PA3626_at | PA3626 |  |  | 2.031 | 0.00129 | conserved hypothetical protein |  | Hypothetical, unclassified, unknown |
| PA3634_at | PA3634 |  |  | 2.031 | 0.000269 | conserved hypothetical protein |  | Hypothetical, unclassified, unknown |
| PA0392_at | PA0392 |  |  | 2.019 | 0.0129 | conserved hypothetical protein |  | Hypothetical, unclassified, unknown |
| PA0076_at | PA0076 |  |  | 2.018 | 0.0212 | hypothetical protein |  | Hypothetical, unclassified, unknown |
| PA4321_at | PA4321 |  |  | 2.018 | 0.0477 | hypothetical protein |  | Hypothetical, unclassified, unknown |
| PA0758_at | PA0758 |  |  | 2.014 | 0.002 | hypothetical protein |  | Hypothetical, unclassified, unknown |
| PA0591_at | PA0591 |  |  | 2.01 | 0.0459 | conserved hypothetical protein |  | Hypothetical, unclassified, unknown |
| PA0091_at | PA0091 |  |  | 2.005 | 0.0304 | conserved hypothetical protein |  | Hypothetical, unclassified, unknown |
| PA5340_at | PA5340 |  |  | 2.001 | 0.000578 | hypothetical protein |  | Hypothetical, unclassified, unknown |
| PA4847_accB_at | PA4847 |  |  | 3.055 | 0.0057 | biotin carboxyl carrier protein (BCCP | *acc*B | Fatty acid and phospholipid metabolism |
| PA2966_acpP_at | PA2966 |  |  | 2.547 | 0.000343 | acyl carrier protein | *acp*P | Fatty acid and phospholipid metabolism |
| PA0286_at | PA0286 |  |  | 2.452 | 0.000571 | probable fatty acid desaturase |  | Fatty acid and phospholipid metabolism |
| PA3651_cdsA_at | PA3651 |  |  | 2.431 | 0.0358 | phosphatidate cytidylyltransferase | *cds*A | Fatty acid and phospholipid metabolism |
| PA3639_accA_at | PA3639 |  |  | 2.294 | 0.00362 | acetyl-coenzyme A carboxylase carboxyl transferase (alpha subunit) | *acc*A | Fatty acid and phospholipid metabolism |
| PA5129_grx_at | PA5129 |  |  | 3.855 | 0.0334 | glutaredoxin | *grx* | Energy metabolism |
| PA5555_atpG_at | PA5555 |  |  | 3.527 | 0.0287 | ATP synthase gamma chain | *atp*G | Energy metabolism |
| PA5554_atpD_at | PA5554 |  |  | 3.011 | 0.00384 | ATP synthase beta chain | *atp*D | Energy metabolism |
| PA3621_fdxA_at | PA3621 |  |  | 3.01 | 0.0042 | ferredoxin I | *fdx*A | Energy metabolism |
| PA5559_atpE_at | PA5559 |  |  | 2.734 | 0.0205 | atp synthase C chain | *atp*E | Energy metabolism |
| PA5560_atpB_at | PA5560 |  |  | 2.433 | 0.0148 | ATP synthase A chain | *atp*B | Energy metabolism |
| PA3635_eno_at | PA3635 |  |  | 2.335 | 0.00382 | enolase | *eno* | Energy metabolism |
| PA5440_at | PA5440 |  |  | 2.257 | 0.00991 | probable peptidase |  | Energy metabolism |
| PA2995_nqrE_at | PA2995 |  |  | 2.216 | 0.00617 | Na+-translocating NADH:quinone oxidoreductase subunit Nqr5 | *nqr*E | Energy metabolism |
| PA5553_atpC_at | PA5553 |  |  | 2.175 | 0.00502 | ATP synthase epsilon chain | *atp*C | Energy metabolism |
| PA0362_fdx1_at | PA0362 |  |  | 2.125 | 0.000346 | ferredoxin [4Fe-4S] | *fdx*1 | Energy metabolism |
| PA1600_at | PA1600 |  |  | 2.076 | 0.00306 | probable cytochrome c |  | Energy metabolism |
| PA4723_dksA_at | PA4723 |  |  | 3.029 | 0.0119 | suppressor protein DKsA | *dks*A | DNA replication, recombination, modification and repair |
| PA0003_recF_at | PA0003 |  |  | 2.855 | 0.000529 | RecF protein | *rec*F | DNA replication, recombination, modification and repair |
| PA5320_dfp_at | PA5320 |  |  | 2.376 | 0.00432 | Phosphopantothenoylcysteine synthase/(R)-4'-phospho-N-pantothenoylcysteine decarboxylase | *dfp* | DNA replication, recombination, modification and repair |
| PA3940_at | PA3940 |  |  | 2.364 | 0.00122 | probable DNA binding protein |  | DNA replication, recombination, modification and repair |
| PA4042_xseB_at | PA4042 |  |  | 2.342 | 0.00985 | exodeoxyribonuclease VII small subunit | *xse*B | DNA replication, recombination, modification and repair |
| PA0001_dnaA_at | PA0001 |  |  | 2.258 | 0.00439 | chromosomal replication initiator protein DnaA | *dna*A | DNA replication, recombination, modification and repair |
| PA0004_gyrB_at | PA0004 |  |  | 2.002 | 6.12E-05 | DNA gyrase subunit B | *gyr*B | DNA replication, recombination, modification and repair |
| PA3168_gyrA_at | PA3168 |  |  | 2.002 | 0.0218 | DNA gyrase subunit A | *gyr*A | DNA replication, recombination, modification and repair |
| PA5362_at | PA5362 |  |  | 2.46 | 0.0243 | conserved hypothetical protein |  | conserved hypothetical protein |
| PA0409_pilH_at | PA0409 |  |  | 2.599 | 0.0241 | twitching motility protein PilH | *pil*H | Chemotaxis |
| PA3811_hscB_at | PA3811 |  |  | 2.997 | 0.0171 | heat shock protein HscB | *hsc*B | Chaperones & heat shock proteins |
| PA4572_fklB_at | PA4572 |  |  | 2.688 | 0.0157 | peptidyl-prolyl cis-trans isomerase FklB | *fklB* | Chaperones & heat shock proteins |
| PA4558_at | PA4558 |  |  | 2.573 | 0.0228 | probable peptidyl-prolyl cis-trans isomerase, FkbP-type |  | Chaperones & heat shock proteins |
| PA1802_clpX_at | PA1802 |  |  | 2.137 | 0.0391 | ATP-dependent Clp protease ATP-binding subunit ClpX | *clpX* | Chaperones & heat shock proteins |
| PA3737_dsbC_at | PA3737 |  |  | 2.032 | 0.00148 | thiol:disulfide interchange protein DsbC | *dsb*C | Chaperones & heat shock proteins |
| PA0363_coaD_at | PA0363 |  |  | 2.42 | 0.0495 | phosphopantetheine adenylyltransferase | *coa*D | Central intermediary metabolism |
| PA4031_ppa_at | PA4031 |  |  | 2.277 | 0.00852 | inorganic pyrophosphatase | *ppa* | Central intermediary metabolism |
| PA5435_at | PA5435 |  |  | 2.251 | 0.0345 | probable transcarboxylase subunit |  | Central intermediary metabolism |
| PA3582_glpK_at | PA3582 |  |  | 2.166 | 0.0165 | glycerol kinase | *glp*K | Central intermediary metabolism |
| PA1838_cysI_at | PA1838 |  |  | 2.053 | 0.0387 | sulfite reductase | *cys*I | Central intermediary metabolism |
| PA3337_rfaD_at | PA3337 |  |  | 3.397 | 0.0498 | ADP-L-glycero-D-mannoheptose 6-epimerase | *rfa*D | Cell wall / LPS / capsule |
| PA4450_murA_at | PA4450 |  |  | 2.855 | 0.00044 | UDP-N-acetylglucosamine 1-carboxyvinyltransferase | *mur*A | Cell wall / LPS / capsule |
| PA3644_lpxA_at | PA3644 |  |  | 2.83 | 0.00038 | UDP-N-acetylglucosamine acyltransferase | *lpx*A | Cell wall / LPS / capsule |
| PA5276_lppL_i_at | PA5276 |  |  | 2.786 | 0.0149 | Lipopeptide LppL precursor | *lpp*L | Cell wall / LPS / capsule |
| PA3643_lpxB_at | PA3643 |  |  | 2.561 | 0.0247 | lipid A-disaccharide synthase | *lpx*B | Cell wall / LPS / capsule |
| PA4545_comL_at | PA4545 |  |  | 2.49 | 0.0182 | competence protein ComL | *com*L | Cell wall / LPS / capsule |
| PA4997_msbA_at | PA4997 |  |  | 2.192 | 0.0133 | transport protein MsbA | *msb*A | Cell wall / LPS / capsule |
| PA5012_waaF_at | PA5012 |  |  | 2.092 | 0.00978 | heptosyltransferase II | *waa*F | Cell wall / LPS / capsule |
| PA1800_tig_at | PA1800 |  |  | 3.266 | 0.00992 | trigger factor | *tig* | Cell division |
| PA4480_mreC_at | PA4480 |  |  | 2.561 | 0.0039 | rod shape-determining protein MreC | *mre*C | Cell division |
| PA5563_soj_at | PA5563 |  |  | 2.456 | 0.00784 | chromosome partitioning protein Soj | *soj* | Cell division |
| PA4481_mreB_at | PA4481 |  |  | 2.304 | 0.00981 | rod shape-determining protein MreB | *mre*B | Cell division |
| PA5562_spoOJ_at | PA5562 |  |  | 2.222 | 0.00304 | chromosome partitioning protein Spo0J | *spo*OJ | Cell division |
| PA1602_at | PA1602 |  |  | 3.119 | 0.00498 | Probable oxidoreductase |  | Carbon compound catabolism |
| PA3183_zwf_at | PA3183 |  |  | 3.109 | 0.00508 | glucose-6-phosphate 1-dehydrogenase | *zwf* | Carbon compound catabolism |
| PA5110_fbp_at | PA5110 |  |  | 2.996 | 0.0273 | fructose-1,6-bisphosphatase | *fbp* | Carbon compound catabolism |
| PA5332_crc_at | PA5332 |  |  | 2.17 | 0.0463 | catabolite repression control protein | *crc* | Carbon compound catabolism |
| PA5192_pckA_at | PA5192 |  |  | 2.151 | 0.042 | phosphoenolpyruvate carboxykinase | *pck*A | Carbon compound catabolism |
| PA3652_uppS_at | PA3652 |  |  | 2.508 | 0.0258 | undecaprenyl pyrophosphate synthetase | *upp*S | Biosynthesis of cofactors, prosthetic groups and carriers |
| PA0381_thiG_at | PA0381 |  |  | 2.498 | 0.0473 | thiamine biosynthesis protein, thiazole moiety | *thi*G | Biosynthesis of cofactors, prosthetic groups and carriers |
| PA0342_thyA_at | PA0342 |  |  | 2.184 | 0.0176 | thymidylate synthase | *thy*A | Biosynthesis of cofactors, prosthetic groups and carriers |
| PA4750_folP_at | PA4750 |  |  | 2.1 | 0.00151 | dihydropteroate synthase | *fol*P | Biosynthesis of cofactors, prosthetic groups and carriers |
| PA4561_ribF_at | PA4561 |  |  | 2.023 | 0.00763 | riboflavin kinase/FAD synthase | *rib*F | Biosynthesis of cofactors, prosthetic groups and carriers |
| PA4569_ispB_at | PA4569 |  |  | 2.877 | 0.00296 | octaprenyl-diphosphate synthase | *isp*B | Biosynthesis of cofactors |
| PA4602_glyA3_at | PA4602 |  |  | 3.347 | 2.83E-05 | serine hydroxymethyltransferase | *gly*A3 | Amino acid biosynthesis and metabolism |
| PA3987_leuS_at | PA3987 |  |  | 2.632 | 0.0216 | leucyl-tRNA synthetase | *leu*S | Amino acid biosynthesis and metabolism |
| PA0009_glyQ_at | PA0009 |  |  | 2.568 | 0.000361 | glycyl-tRNA synthetase alpha chain | *gly*Q | Amino acid biosynthesis and metabolism |
| PA4443_cysD_at | PA4443 |  |  | 2.464 | 0.00794 | ATP sulfurylase small subunit | *cys*D | Amino acid biosynthesis and metabolism |
| PA3525_argG_at | PA3525 |  |  | 2.45 | 0.0411 | argininosuccinate synthase | *arg*G | Amino acid biosynthesis and metabolism |
| PA3167_serC_at | PA3167 |  |  | 2.345 | 0.0396 | 3-phosphoserine aminotransferase | *ser*C | Amino acid biosynthesis and metabolism |
| PA0904_lysC_at | PA0904 |  |  | 2.337 | 0.00528 | aspartate kinase alpha and beta chain | *lys*C | Amino acid biosynthesis and metabolism |
| PA5263_argH_at | PA5263 |  |  | 2.29 | 0.00853 | argininosuccinate lyase | *arg*H | Amino acid biosynthesis and metabolism |
| PA4007_proA_at | PA4007 |  |  | 2.282 | 0.0231 | gamma-glutamyl phosphate reductase | *pro*A | Amino acid biosynthesis and metabolism |
| PA5277_lysA_at | PA5277 |  |  | 2.247 | 0.0334 | diaminopimelate decarboxylase | *lys*A | Amino acid biosynthesis and metabolism |
| PA5143_hisB_at | PA5143 |  |  | 2.246 | 0.00461 | imidazoleglycerol-phosphate dehydratase | *his*B | Amino acid biosynthesis and metabolism |
| PA5039_aroK_at | PA5039 |  |  | 2.234 | 0.013 | shikimate kinase | *aro*K | Amino acid biosynthesis and metabolism |
| PA0018_fmt_at | PA0018 |  |  | 2.222 | 0.0137 | methionyl-tRNA formyltransferase | *fmt* | Amino acid biosynthesis and metabolism |
| PA0649_trpG_at | PA0649 |  |  | 2.212 | 0.0118 | anthranilate synthase component II | *trp*G | Amino acid biosynthesis and metabolism |
| PA5067_hisE_at | PA5067 |  |  | 2.212 | 0.00214 | phosphoribosyl-ATP pyrophosphohydrolase | *his*E | Amino acid biosynthesis and metabolism |
| PA0402_pyrB_at | PA0402 |  |  | 2.185 | 0.0354 | aspartate carbamoyltransferase | *pyr*B | Amino acid biosynthesis and metabolism |
| PA2253_ansA_at | PA2253 |  |  | 2.185 | 0.0471 | L-asparaginase I | *ans*A | Amino acid biosynthesis and metabolism |
| PA1687_speE_at | PA1687 |  |  | 2.148 | 0.000393 | spermidine synthase | *spe*E | Amino acid biosynthesis and metabolism |
| PA3108_purF_at | PA3108 |  |  | 2.09 | 0.0294 | amidophosphoribosyltransferase | *pur*F | Amino acid biosynthesis and metabolism |
| PA5203_gshA_at | PA5203 |  |  | 2.088 | 0.0259 | glutamate--cysteine ligase | *gsh*A | Amino acid biosynthesis and metabolism |
| PA3482_metG_at | PA3482 |  |  | 2.043 | 0.0312 | methionyl-tRNA synthetase | *met*G | Amino acid biosynthesis and metabolism |
| PA5119_glnA_at | PA5119 |  |  | 2.038 | 0.0442 | glutamine synthetase | *gln*A | Amino acid biosynthesis and metabolism |
| PA4439_trpS_at | PA4439 |  |  | 2.002 | 0.0155 | tryptophanyl-tRNA synthetase | *trp*S | Amino acid biosynthesis and metabolism |
| PA4671_at | PA4671 |  |  | 5.186 | 0.0243 | 50 S ribosomal protein L25 |  | Adaptation, protection |
| PA0594_surA_at | PA0594 |  |  | 2.606 | 0.00348 | peptidyl-prolyl cis-trans isomerase | *sur*A | Adaptation, protection |
| PA4760_dnaJ_at | PA4760 |  |  | 2.554 | 0.000159 | DnaJ protein | *dna*J | Adaptation, protection |
| PA0595_ostA_at | PA0595 |  |  | 2.383 | 0.019 | organic solvent tolerance protein OstA precursor | *ost*A | Adaptation, protection |
| PA4428_sspA_at | PA4428 |  |  | 2.282 | 0.00718 | stringent starvation protein A | *ssp*A | Adaptation, protection |
| PA1803_lon_at | PA1803 |  |  | 2.023 | 0.0478 | Lon protease | *lon* | Adaptation, protection |
| PA5568_at | PA5568 |  |  | 2.656 | 0.0116 | conserved hypothetical protein |  | Membrane proteins |
| PA4317_at | PA4317 |  |  | 2.636 | 0.0148 | hypothetical protein |  | Membrane proteins |
| PA3665_at | PA3665 |  |  | 2.414 | 0.000496 | hypothetical protein |  | Membrane proteins |
| PA3747_at | PA3747 |  |  | 2.361 | 0.0142 | conserved hypothetical protein |  | Membrane proteins |
| PA1774_at | PA1774 |  |  | 2.142 | 0.041 | hypothetical protein |  | Membrane proteins |
| **Group V: No change(20min) - Downregulation(60 min) 70 genes** | | | | | | | | |
| PA3038_at | PA3038 |  |  | -2.067 | 0.035 | probable porin |  | Transport of small molecules |
| PA1871_lasA_at | PA1871 |  |  | -7.299 | 0.00137 | LasA protease precursor | *Las*A | Translation, post-translational modification, degradation |
| PA0797_at | PA0797 |  |  | -2.037 | 0.0303 | probable transcriptional regulator |  | Transcriptional regulators |
| PA4296_at | PA4296 |  |  | -3.802 | 0.00373 | probable two-component response regulator |  | Transcriptional regulators |
| PA1250_aprI_at | PA1250 |  |  | -2.208 | 0.0198 | alkaline proteinase inhibitor AprI | *apr*I | Secreted Factors (toxins, enzymes, alginate) |
| PA1905_s_at | PA1905 |  |  | -5.78 | 0.0125 | probable pyridoxamine 5'-phosphate oxidase |  | Secreted Factors (toxins, enzymes, alginate) |
| PA0130_at | PA0130 |  |  | -2.299 | 0.0061 | probable aldehyde dehydrogenase |  | Putative enzymes |
| PA0656_at | PA0656 |  |  | -2.488 | 0.00566 | probable HIT family protein |  | Putative enzymes |
| PA2815_at | PA2815 |  |  | -2.659 | 0.0275 | probable acyl-CoA dehydrogenase |  | Putative enzymes |
| PA3723_at | PA3723 |  |  | -2.801 | 0.047 | probable FMN oxidoreductase |  | Putative enzymes |
| PA2552_at | PA2552 |  |  | -3.623 | 0.012 | probable acyl-CoA dehydrogenase |  | Putative enzymes |
| PA2013_at | PA2013 |  |  | -4.629 | 0.0202 | probable enoyl-CoA hydratase/isomerase |  | Putative enzymes |
| PA0745_at | PA0745 |  |  | -5.291 | 0.00721 | probable enoyl-CoA hydratase/isomerase |  | Putative enzymes |
| PA1984_s_at | PA1984 |  |  | -5.848 | 0.0199 | probable aldehyde dehydrogenase |  | Putative enzymes |
| PA4217_at | PA4217 |  |  | -6.623 | 0.00936 | Probable FAD-dependent monooxygenase |  | Putative enzymes |
| PA2574_at | PA2574 |  |  | -3.175 | 0.0428 | conserved hypothetical protein |  | Membrane proteins |
| PA1041_at | PA1041 |  |  | -6.536 | 0.000705 | probable outer membrane protein |  | Membrane proteins |
| PA4300_at | PA4300 |  |  | -2.976 | 0.00857 | hypothetical protein |  | Membrane proteins |
| PA2779_at | PA2779 |  |  | -2.037 | 0.0239 | hypothetical protein |  | Hypothetical, unclassified, unknown |
| PA3986_at | PA3986 |  |  | -2.04 | 0.000774 | hypothetical protein |  | Hypothetical, unclassified, unknown |
| PA3945_at | PA3945 |  |  | -2.088 | 0.0419 | conserved hypothetical protein |  | Hypothetical, unclassified, unknown |
| PA2816_i_at | PA2816 |  |  | -2.092 | 0.0125 | hypothetical protein |  | Hypothetical, unclassified, unknown |
| PA3089_at | PA3089 |  |  | -2.123 | 0.000869 | hypothetical protein |  | Hypothetical, unclassified, unknown |
| PA1784_at | PA1784 |  |  | -2.336 | 0.012 | hypothetical protein |  | Hypothetical, unclassified, unknown |
| PA2504_at | PA2504 |  |  | -2.364 | 0.0368 | hypothetical protein |  | Hypothetical, unclassified, unknown |
| PA1732_at | PA1732 |  |  | -2.375 | 0.0107 | conserved hypothetical protein |  | Hypothetical, unclassified, unknown |
| PA2031_i_at | PA2031 |  |  | -2.387 | 0.0156 | hypothetical protein |  | Hypothetical, unclassified, unknown |
| PA2009_hmgA_at | PA5359 |  |  | -2.469 | 0.00567 | hypothetical protein |  | Hypothetical, unclassified, unknown |
| PA1348_at | PA1348 |  |  | -2.538 | 0.0101 | hypothetical protein |  | Hypothetical, unclassified, unknown |
| PA4311_at | PA4311 |  |  | -2.646 | 0.0386 | conserved hypothetical protein |  | Hypothetical, unclassified, unknown |
| PA5178_at | PA5178 |  |  | -2.688 | 0.0275 | conserved hypothetical protein |  | Hypothetical, unclassified, unknown |
| PA0960_at | PA0960 |  |  | -2.695 | 0.00967 | hypothetical protein |  | Hypothetical, unclassified, unknown |
| PA5460_at | PA5460 |  |  | -2.71 | 0.0281 | hypothetical protein |  | Hypothetical, unclassified, unknown |
| PA4925_at | PA4925 |  |  | -2.732 | 0.0315 | conserved hypothetical protein |  | Hypothetical, unclassified, unknown |
| PA3123_at | PA3250 |  |  | -2.865 | 0.0114 | hypothetical protein |  | Hypothetical, unclassified, unknown |
| PA3250_at | PA3123 |  |  | -2.8658 | 0.0212 | conserved hypothetical protein |  | Hypothetical, unclassified, unknown |
| PA4874_at | PA4874 |  |  | -2.924 | 0.011 | hypothetical protein |  | Hypothetical, unclassified, unknown |
| PA4299_at | PA4299 |  |  | -3.03 | 0.000994 | hypothetical protein |  | Hypothetical, unclassified, unknown |
| PA3923_at | PA3923 |  |  | -3.333 | 0.00414 | hypothetical protein |  | Hypothetical, unclassified, unknown |
| PA3361_at | PA3361 |  |  | -3.413 | 0.00691 | hypothetical protein |  | Hypothetical, unclassified, unknown |
| PA2367_at | PA2367 |  |  | -3.584 | 0.0244 | hypothetical protein |  | Hypothetical, unclassified, unknown |
| PA0852_cpbD_at | PA0852 |  |  | -3.61 | 0.0333 | chitin-binding protein CbpD precursor | *cpb*D | Hypothetical, unclassified, unknown |
| PA0587_at | PA0587 |  |  | -4.425 | 0.00404 | conserved hypothetical protein |  | Hypothetical, unclassified, unknown |
| PA0588_at | PA0588 |  |  | -4.808 | 0.0417 | conserved hypothetical protein |  | Hypothetical, unclassified, unknown |
| PA1216_at | PA1216 |  |  | -5.917 | 0.0293 | hypothetical protein |  | Hypothetical, unclassified, unknown |
| PA2015_at | PA2015 |  |  | -4.629 | 0.0105 | Citronelloyl-CoA dehydrogenase, GnyD |  | Fatty acid and phospholipid metabolism |
| PA3417_at | PA3417 |  |  | -2.817 | 0.00349 | probable pyruvate dehydrogenase E1 component, alpha subunit |  | Energy metabolism |
| PA1175_napD_at | PA1175 |  |  | -2.949 | 0.0134 | NapD protein of periplasmic nitrate reductase | *nap*D | Energy metabolism |
| PA1176_napF_at | PA1176 |  |  | -2.949 | 0.024 | ferredoxin protein NapF | *nap*F | Energy metabolism |
| PA1173_napB_at | PA1173 |  |  | -3.077 | 0.00344 | cytochrome c-type protein NapB precursor | *nap*B | Energy metabolism |
| PA0107_at | PA0107 |  |  | -3.425 | 0.0192 | hypothetical |  | Energy metabolism |
| PA1174_napA_at | PA1174 |  |  | -3.425 | 0.0251 | periplasmic nitrate reductase protein NapA | *nap*A | Energy metabolism |
| PA1562_acnA_at | PA1562 |  |  | -3.571 | 0.0342 | aconitate hydratase 1 | *acn*A | Energy metabolism |
| PA5058_phaC2_at | PA5058 |  |  | -2.217 | 0.0216 | poly(3-hydroxyalkanoic acid) synthase 2 | *pha*C2 | Central intermediary metabolism |
| PA5355_glcD_at | PA5355 |  |  | -2.457 | 0.0319 | glycolate oxidase subunit GlcD | *glc*D | Central intermediary metabolism |
| PA5359_at | PA2009 |  |  | -2.469 | 0.00399 | homogentisate 1,2-dioxygenase | *hmg*A | Carbon compound catabolism |
| PA4590_pra_at | PA4590 |  |  | -2.558 | 0.00383 | protein activator | *pra* | Carbon compound catabolism |
| PA0792_prpD_at | PA0792 |  |  | -3.378 | 0.00581 | propionate catabolic protein PrpD | *prp*D | Carbon compound catabolism |
| PA3570_mmsA_at | PA3570 |  |  | -3.021 | 0.000376 | methylmalonate-semialdehyde dehydrogenase | *mms*A | Amino acid biosynthesis and metabolism |
| PA3418_ldh_at | PA3418 |  |  | -3.236 | 0.0102 | leucine dehydrogenase | *ldh* | Amino acid biosynthesis and metabolism |
| PA2250_lpdV_at | PA2250 |  |  | -3.497 | 0.00576 | lipoamide dehydrogenase-Val | *lpd*V | Amino acid biosynthesis and metabolism |
| PA0132_at | PA0132 |  |  | -3.69 | 0.00487 | beta-alanine--pyruvate transaminase |  | Amino acid biosynthesis and metabolism |
| PA2249_bkdB_at | PA2249 |  |  | -4.785 | 0.0415 | branched-chain alpha-keto acid dehydrogenase (lipoamide component) | *bkd*B | Amino acid biosynthesis and metabolism |
| PA2248_bkdA2_at | PA2248 |  |  | -5.076 | 0.0072 | 2-oxoisovalerate dehydrogenase (beta subunit) | *bkd*A2 | Amino acid biosynthesis and metabolism |
| PA2247_bkdA1_at | PA2247 |  |  | -9.434 | 0.00376 | 2-oxoisovalerate dehydrogenase (alpha subunit) | *bkd*A1 | Amino acid biosynthesis and metabolism |
| PA2573_at | PA2573 |  |  | -2.597 | 0.019 | probable chemotaxis transducer |  | Adaptation, protection; Chemotaxis |
| PA0175_at | PA0175 |  |  | -2.02 | 0.0449 | probable chemotaxis protein methyltransferase |  | Adaptation, protection |
| PA2920_at | PA2920 |  |  | -2.232 | 0.00588 | probable chemotaxis transducer |  | Adaptation, protection |
| PA4614_mscL_at | PA4614 |  |  | -2.278 | 0.00914 | conductance mechanosensitive channel | *msc*L | Adaptation, protection |
| PA2622_cspD_at | PA2622 |  |  | -3.597 | 0.00599 | cold-shock protein CspD | *csp*D | Adaptation, protection |
| **Group VI: Downregulation (20min) - Downregulation (60 min) 26 genes** | | | | | | | | |
| PA1342_at | PA1342 | -2.288 | 0.0253 | -3.802 | 0.0253 | probable binding protein component of ABC transporter |  | Transport of small molecules |
| PA3049_rmf_at | PA3049 | -6.25 | 0.000723 | -25.907 | 0.000723 | ribosome modulation factor | *rmf* | Translation, post-translational modification, degradation |
| PA3724_lasB_at | PA3724 | -2.762 | 0.00182 | -10.256 | 0.00182 | elastase LasB | *las*B | Translation, post-translational modification, degradation |
| PA3622_rpoS_at | PA3622 | -2.653 | 0.00961 | -2.967 | 0.00961 | sigma factor RpoS | *rpo*S | Transcriptional regulators |
| PA4211_g_at | PA4211 | -3.448 | 0.0171 | -15.923 | 0.0171 | probable phenazine biosynthesis protein |  | Secreted Factors (toxins, enzymes, alginate) |
| PA5445_at | PA5445 | -8.547 | 0.000178 | -3.174 | 0.000178 | probable coenzyme A transferase |  | Putative enzymes |
| PA2634_at | PA2634 | -2.481 | 0.017 | -2.105 | 0.017 | probable isocitrate lyase |  | Putative enzymes |
| PA0998_at | PA0998 | -2.123 | 0.0424 | -2.016 | 0.0424 | hypothetical protein |  | Putative enzymes |
| PA3720_at | PA3720 | -10.672 | 0.0037 | -3.012 | 0.0037 | hypothetical protein |  | Hypothetical, unclassified, unknown |
| PA3496_at | PA3496 | -2.488 | 0.0104 | -2.183 | 0.0104 | hypothetical protein |  | Hypothetical, unclassified, unknown |
| PA2381_at | PA2381 | -2.475 | 0.0344 | -4.651 | 0.0344 | hypothetical protein |  | Hypothetical, unclassified, unknown |
| PA4607_at | PA4607 | -2.381 | 0.00355 | -11.013 | 0.00355 | hypothetical protein |  | Hypothetical, unclassified, unknown |
| PA3205_at | PA3205 | -2.132 | 0.00392 | -3.14 | 0.00392 | hypothetical protein |  | Hypothetical, unclassified, unknown |
| PA4133_at | PA4133 | -2.625 | 0.0376 | -7.463 | 0.0376 | cytochrome c oxidase subunit (cbb3-type) |  | Energy metabolism |
| PA4762_grpE_at | PA4762 | -2.915 | 0.00501 | -0.479 | 0.00501 | heat shock protein GrpE | *grp*E | DNA replication, recombination, modification and repair |
| PA5054_hslU_at | PA5054 | -3.226 | 0.000316 | -0.428 | 0.000316 | heat shock protein HslU | *hsl*U | Chaperones & heat shock proteins |
| PA1596_htpG_at | PA1596 | -2.695 | 0.00706 | -0.434 | 0.00706 | heat shock protein HtpG | *htp*G | Chaperones & heat shock proteins |
| PA5053_hslV_at | PA5053 | -2.597 | 0.00113 | -0.352 | 0.00113 | heat shock protein HslV | *hsl*V | Chaperones & heat shock proteins |
| PA0482_glcB_at | PA0482 | -4.405 | 0.00719 | -3.584 | 0.00719 | malate synthase G | *glc*B | Carbon compound catabolism; |
| PA0887_acsA_at | PA0887 | -7.246 | 0.00233 | -10.04 | 0.00233 | acetyl-coenzyme A synthetase | *acs*A | Carbon compound catabolism |
| PA0795_prpC_at | PA0795 | -3.333 | 0.0112 | -5.236 | 0.0112 | citrate synthase 2 | *prp*C | Carbon compound catabolism |
| PA0796_prpB_at | PA0796 | -2.825 | 0.0376 | -4.219 | 0.0376 | carboxyphosphonoenolpyruvate phosphonomutase | *prp*B | Carbon compound catabolism |
| PA1986_pqqB_at | PA1986 | -4.167 | 0.00323 | -3.165 | 0.00323 | pyrroloquinoline quinone biosynthesis protein B | *pqq*B | Biosynthesis of cofactors, prosthetic groups and carriers |
| PA2623_icd_at | PA2623 | -2.457 | 0.0191 | -2.558 | 0.0191 | isocitrate dehydrogenase | *icd* | Amino acid biosynthesis and metabolism |
| PA4290_at | PA4290 | -2.874 | 0.0455 | -2.778 | 0.0455 | probable chemotaxis transducer |  | Adaptation, protection |
| PA4761_dnaK_at | PA4761 | -2.786 | 0.000515 | -0.404 | 0.000515 | DnaK protein | *Dna*K | Adaptation, protection |
